# Population genomics reveals lack of greater white-fronted introgression into the Swedish lesser white-fronted goose

**DOI:** 10.1038/s41598-020-75315-y

**Published:** 2020-10-27

**Authors:** David Díez-del-Molino, Johanna von Seth, Niclas Gyllenstrand, Fredrik Widemo, Niklas Liljebäck, Mikael Svensson, Per Sjögren-Gulve, Love Dalén

**Affiliations:** 1 Department of Bioinformatics and Genetics, Swedish Museum of Natural History, Box 50007, 10405 Stockholm, Sweden; 2 Department of Zoology, Stockholm University, 10691 Stockholm, Sweden; 3 Centre for Genetic Identification, Department of Environmental Research and Monitoring, Swedish Museum of Natural History, Box 50007, 10405 Stockholm, Sweden; 4 Department of Wildlife, Fish and Environmental Studies, Swedish University of Agricultural Sciences, 90183 Umeå, Sweden; 5 Grimsö Wildlife Research Station, Department of Ecology, Swedish University of Agricultural Sciences, 73091 Riddarhyttan, Sweden; 6 Swedish Species Information Centre, SLU ArtDatabanken, Box 7007, 750 07 Uppsala, Sweden; 7 The Wildlife Analysis Unit, Swedish Environmental Protection Agency, 106 48 Stockholm, Sweden

**Keywords:** Evolutionary genetics, Population genetics, Conservation genomics, Genetic hybridization

## Abstract

Interspecific introgression is considered a potential threat to endangered taxa. One example where this has had a major impact on conservation policy is the lesser white-fronted goose (LWfG). After a dramatic decline in Sweden, captive breeding birds were released between 1981–1999 with the aim to reinforce the population. However, the detection of greater white-fronted goose (GWfG) mitochondrial DNA in the LWfG breeding stock led to the release program being dismantled, even though the presence of GWfG introgression in the actual wild Swedish LWfG population was never documented. To examine this, we sequenced the complete genomes of 21 LWfG birds from the Swedish, Russian and Norwegian populations, and compared these with genomes from other goose species, including the GWfG. We found no evidence of interspecific introgression into the wild Swedish LWfG population in either nuclear genomic or mitochondrial data. Moreover, Swedish LWfG birds are genetically distinct from the Russian and Norwegian populations and display comparatively low genomic diversity and high levels of inbreeding. Our findings highlight the utility of genomic approaches in providing scientific evidence that can help improve conservation management as well as policies for breeding and reinforcement programmes.

## Introduction

Interspecific introgression has often been highlighted as a potential threat to endangered taxa due to the risk of outbreeding depression^1^. The reason for this is that the introduction of genes from a different taxon into a small threatened population can reduce the fitness of hybrids^1,2^. This has led to introgressed individuals being identified as a cause for concern during translocation or reinforcement actions^3^. At a genetic level, the mechanisms behind outbreeding depression include breakup of coadapted gene complexes and disruption of local adaptations. For example, the introduction of Middle Eastern Ibex individuals to reinforce the Alpine Ibex population in Czechoslovakia resulted in calves being born in the middle of winter, which led to the subsequent disappearance of the Czech population^4^. In general, when captive populations have been identified as containing hybrids, the management recommendation has often been to exclude these individuals as potential sources for reinforcement programmes^5–7^.

**Figure 1:**
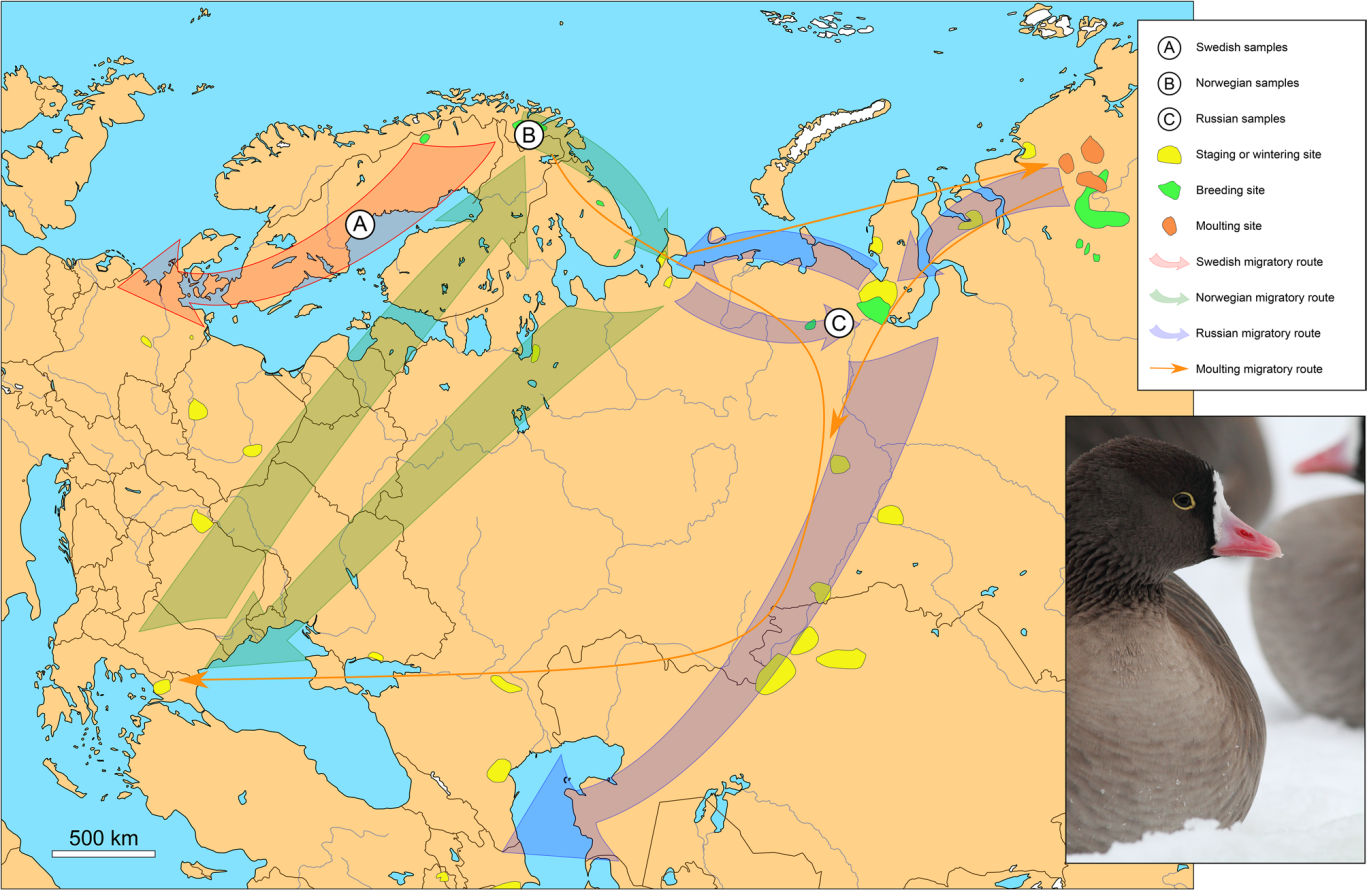
Geographical location of sampling sites for the lesser white-fronted goose. Sampling sites are denoted with white circles. Important sites and main migratory routes for the Western Palearctic LWfG are also depicted (Adapted from Aarvak et al.^64^). Norwegian migratory routes represent the Fennoscandian population routes; Russian, the Western Main population routes; and Swedish, the westward modified migratory route from the 1989–1999 reinforcement program in Sweden. Map was downloaded from Natural Earth (naturalearthdata.com) and edited in QGIS v2.18.17 (qgis.org) and Inkscape v0.92 (inkscape.org).

One particular case where concerns about interspecific introgression have had a major impact on conservation policy is the lesser white-fronted goose (*Anser erythropus,* LWfG) population in Sweden^8^. Until the early 1900s, the LWfG was a widespread and relatively common breeding bird in arctic and semi-arctic areas of northern Eurasia (e.g. Madsen & Cracknell^9^). Historically, three different populations, mainly delimited by migration routes and breeding areas, have been identified: the Fennoscandian, the Western Main, and the Eastern Main populations (Fig. 1). During the 20th Century, the Fennoscandian population suffered a dramatic decline to the extent that in the 1980s only 60–90 breeding pairs remained in two small populations, one in Norway and another one in Sweden, with only 20 pairs in the Swedish mountain tundra^10,11^. This dramatic decline has mainly been explained by increased mortality due to overhunting during migration and in wintering grounds (e.g. Madsen & Cracknell^9^).

As a response to the dwindling population numbers, in 1977 the Swedish Association for Hunting and Wildlife Management initiated an ex-situ breeding programme with the objective to release birds to reinforce the Swedish LWfG population^12^. The exact origin of the founder birds of this programme is obscure, but it is known that it included wild-caught birds from Swedish Lapland^13^ as well as captive birds from parks in the Netherlands and England^14^. From 1981 to 1999, a total of 341 birds were released into an area that was known to hold native LWfG breeding pairs prior to the start of the reinforcement programme^11,15^.

In the late 1990s, genetic studies suggested that the reinforcement programme may have inadvertently introduced LWfG individuals carrying greater white-fronted goose (*Anser albifrons,* GWfG) genes into the LWfG Swedish population. Based on short mitochondrial DNA fragments (~ 200 bp), 16% of the adult LWfG captive population was identified as carrying GWfG haplotypes^16^. Consequently, the original Swedish captive breeding programme was dismantled in 2000 as a precautionary measure. Unfortunately, the juvenile goslings that had been released into the wild were never genetically tested, so the real amount of introgressed individuals released is unknown. An estimate using parentage analysis based on archival data of the breeding project suggested that 5–10% of the released juveniles may have contained GWfG introgressed genes^8^. Following this, no subsequent genetic studies have been done to investigate the genetic composition and levels of introgression of the present-day LWfG Swedish population. However, inferences of genetic relationships and gene flow based on short mitochondrial DNA sequences can sometimes be problematic. This is due not only to their low resolution in phylogenetic analyses, but also their sensitivity to incomplete lineage sorting. Moreover, previous studies have also indicated a high prevalence of nuclear insertions of mitochondrial DNA (numts) in *Anser sp.*^1,7^, which can further complicate the inferences.

During the last decade, major technological developments in DNA sequencing technology have made it feasible to sequence complete nuclear genomes at reasonable economic costs^1,8^. Such whole genome data has revolutionized the possibility to identify introgression among species^19–21^. Together with genomic data, new computational approaches have enabled detection of hybridization even when only a small proportion of the genome has a different ancestry^22,23^.

The principal aim of this study was to investigate the existence and degree of introgression from GWfG into the wild Swedish LWfG population. To do this, we sequenced 21 high-coverage genomes from LWfG individuals originating from Sweden, Norway and Russia (Table 1, Fig. 1). We then used this dataset combined with genomes from 18 other goose species (Table S1, Supplementary Information 1.3) and several computational approaches to test for introgression. We also examined the amount of genetic differentiation among the Swedish, Norwegian and Russian populations and estimated the levels of genome-wide diversity and inbreeding within each population. Based on the available data from previous studies, and the fact that seven LWfG generations had passed since the release of putatively hybrid geese and when our samples were collected, we predicted a high prevalence of individuals introgressed with GWfG genes in the LWfG Swedish population. Moreover, we expected that such introgression would have resulted in a higher genome-wide diversity and lower inbreeding levels in the Swedish birds compared to the Norwegian and Russian populations.

**Table 1:** List of LWfG samples newly sequenced in this study.

Sample ID	Lab ID	Origin	Year sampled	Inferred sex	Sequencing effort	Coverage (PfG)	Coverage (Mallard Duck)
SWE01	P7752_101	Swedish	2010	M	80.5	15.72	13.4
SWE02	P7752_102	Swedish	2010	M	78.92	12.05	10.23
SWE03	P7752_103	Swedish	2010	F	79.65	13.5	11.54
SWE04	P7752_104	Swedish	2010	M	81.27	12.21	10.44
SWE05	P7752_105	Swedish	2010	M	79	13.69	11.65
SWE06	P7752_106	Swedish	2010	M	80.97	12.39	10.59
SWE07	P7752_107	Swedish	2010	F	80.3	16.59	14.13
SWE08	P7752_108	Swedish	2010	F	80.41	14.5	12.35
SWE09	P7752_109	Swedish	2010	M	77.9	10.25	8.69
SWE10	P7752_110	Swedish	2010	F	78.48	18.57	15.83
SWE11	P7752_111	Swedish	2015	F	78.84	16.1	13.76
SWE12	P7752_112	Swedish	2016	F	79.99	15.7	13.43
RUS13	P7752_113	Russian	2007	F	79.18	14.24	12.15
RUS14	P7752_114	Russian	2009	F	77.84	16.59	14.13
RUS15	P7752_115	Russian	2007	M	79.28	25.45	21.82
RUS16	P7752_116	Russian	2009	M	81.75	14.32	12.28
RUS17	P7752_117	Russian	2010	F	78.22	15	12.72
NOR19	P7752_119	Norwegian	2000	F	79.75	21.25	18.05
NOR20	P7752_120	Norwegian	2000	F	79.87	17.61	15.03
NOR21	P7752_121	Norwegian	2000	M	79.28	21.73	18.47
NOR22	P7752_122	Norwegian	2000	M	78.7	21.44	18.19

Per sample sequencing effort (in millions of reads) and average genomic coverage (x, after filtering by MQ > 30) when mapped to PfG and the mallard duck are detailed.

## Results

Sequencing yielded an average of 79 million reads per sample after filtering by quality (Table 1). Of those, an average of 94.87% mapped to the pink-footed goose (*Anser brachyrhynchus*, PfG) assembly and an average of 83.86% mapped to the more distantly related mallard duck (*Anas platyrhynchos*). Overall, the newly sequenced genomes had an average coverage of 16.1× (range 10.3–25.5) for the dataset mapped to the PfG, and an average of 13.8× (range 8.7–21.8) for the dataset mapped to the mallard duck. For the goose samples downloaded from Ottenburghs et al.^24,25^ and remapped, the average coverage was 15.3 × when mapped to PfG and 12.4 × when mapped to the mallard duck (Table S1).

RepeatMasker indicated that up to 7.28% of the PfG assembly was composed from highly repetitive regions (Table S2). After excluding these regions and filtering by read depth (≥ 7), allelic balance (> 0.2 and < 0.8), mapping (> 30) and base quality (> 30), variant calling of all 42 individuals yielded 29,023,687 variable sites (Supplementary Information 1.4). For population genetic analyses of LWfG samples, these sites were linkage-disequilibrium pruned (window size of 50 kb, step size of 5 kb, and a minimum pairwise correlation (r^2^) of 0.5 for SNPs to be excluded) leaving a final dataset comprising 6,509,393 variants.

**Figure 2:**
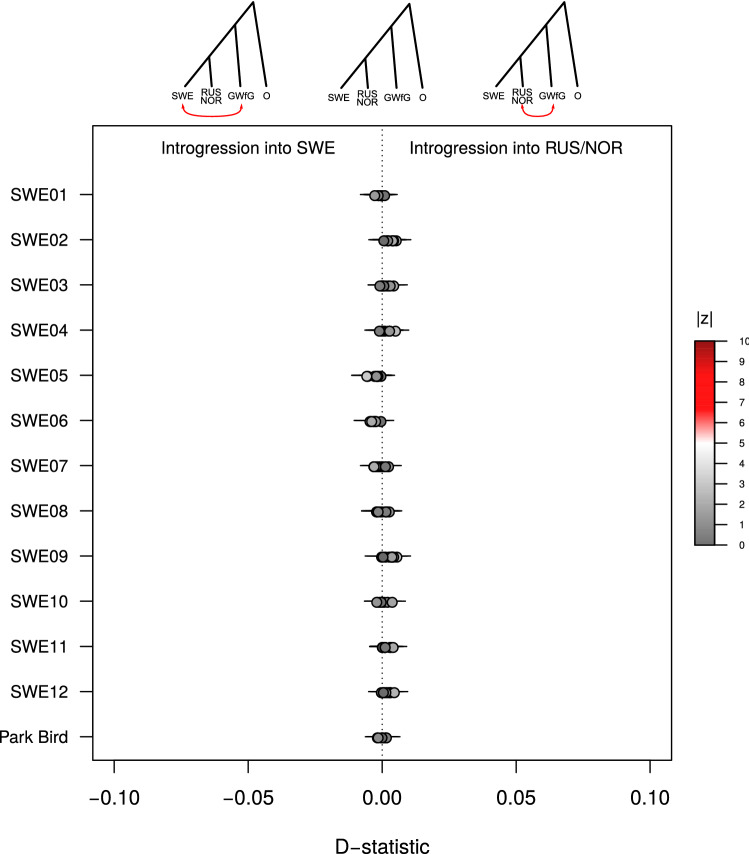
Estimates of introgression from greater white-fronted goose into Swedish respect to Russian and Norwegian lesser white-fronted goose individuals using the pink-footed goose as outgroup. The tests are in the form D(SWE, RUS/NOR; GWfG, PfG). Error bars depicting 3*SE are displayed. Dots are colored representing the |Z| value of the comparison.

The D-statistics suggested that the GWfG has not contributed to the Swedish LWfG population’s gene pool relative to the Norwegian or Russian populations (D = 0 for all comparisons, Fig. 2, Figure S14A). We obtained similar results when extending the test using all the other *Anser* species in the dataset as donors, i.e. D(SWE, NOR/RUS; X, O), where X is any of the other *Anser* species candidates to be introgressed into the Swedish population of LWfG, and O is the bar-headed goose (Supplementary Information 2.3.1, Figure S15A). Regardless of the donor assumed, none of the comparisons showed a clear and consistent departure from D = 0, and thus we found no evidence of introgression from any other goose species into the Swedish LWfG relative to Norwegian or Russian populations. To account for any confounding effect of the reference genome used, we repeated all the tests using the dataset mapped to the mallard duck assembly, which yielded very similar results (Figures S13, S14B and S15B).

A key question, given the results from the D-statistics, is whether sequencing 12 genomes is enough to detect hybrids in LWfG samples collected in 2010 and onwards. Taking into account that the reinforcement programme may have resulted in a minimum of 5% of all Swedish LWfG birds carrying some sort of GWfG ancestry^26^, and assuming a generation time of 3–5 years and no selective disadvantage for those birds with hybrid ancestry, the expected proportion of introgressed LWfG individuals at the time of sampling in 2010 is 0.56—0.96 (see Supplementary Information 2.3.2). Using these figures and a hypergeometric distribution^27^, we estimated that the probability of not detecting GWfG ancestry in any of our 10 birds sampled in 2010, from a finite population of 110, is between P < 0.001 and P < 0.0001. Consequently, it is very unlikely that any meaningful GWfG ancestry had gone undetected given our sampling effort.

To further investigate if any of our sampled LWfG carried GWfG mitochondrial DNA, which could indicate introgression or incomplete lineage sorting, we reconstructed the mitogenomes for the LWfG samples. Accurately reconstructing the mitochondrial genomes was challenging due to the apparent presence of widespread nuclear insertions of mitochondrial DNA (i.e. numts, see Supplementary Information 2.3.3). Nonetheless, regardless of whether we excluded or included sites that indicated presence of numts, the resulting phylogenetic trees grouped all analyzed LWfG and GWfG mitogenomes into two reciprocally monophyletic clades (Figure S17), thus demonstrating that none of our sequenced LWfG carried GWfG mitogenomes.

**Figure 3:**
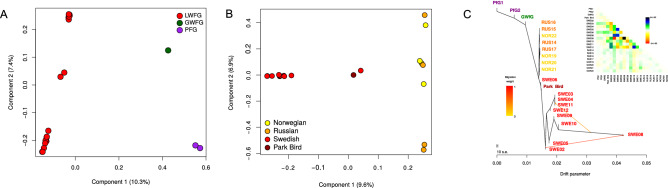
Genetic structure among the lesser white-fronted goose. (**A**) Principal component analysis (PCA) of LWfG, GWfG and PfG samples. (**B**) PCA of the LWfG in this study (excluding first degree relatives). Different colors represent the distinct origins of the samples. (**C**) TREEMIX best model for LWfG (excluding first degree relatives), GWfG and PfG samples in the dataset and two migration edges (m = 2), including the pairwise residuals. ‘Park Bird’, refers to the LWfG sample from Ottenburghs et al.^24,25^.

Principal component analysis (PCA) of the LWfG samples suggested they are structured in two discrete groups, with the majority of Swedish samples in one of them and Norwegian and Russian samples in the other (Fig. 3A,B, Supplementary Information 2.1.3). In fact, the genetic differentiation between these groups is relatively high (Fst = 0.067 SWE-RUS and 0.068 SWE-NOR, Supplementary Information 2.1.4, Table S3). Two Russian and two Swedish samples cluster together and are differentiated from these two main groups. These two pairs were found to be siblings (Figure S3), which is probably affecting their genetic affinities in the PCA (Figure S5) as well as in other analyses based on allele frequencies. Therefore, further analyses were performed removing one individual of each pair of full siblings (see Supplementary Information 2.1.4). Treemix analyses corroborated the PCA results and supported that the Swedish LWfG samples are closely related to each other, but clearly distinct from the group composed of Russian and Norwegian samples (Fig. 3C). The park bird from^24,25^ shows closer genetic affinity to the sibling Swedish LWfG samples in the PCA and the Treemix analyses.

**Figure 4:**
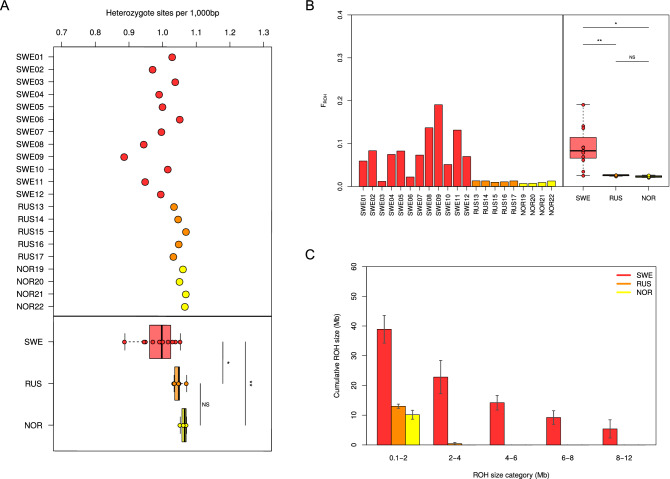
Genetic diversity and inbreeding in lesser white-fronted goose. (**A**) Genome wide levels of diversity estimated as number of heterozygote sites per 1kbp in all LWfG samples. (**B**) Inbreeding values (F_ROH_) for all LWfG birds analyzed in the dataset. (**C**) Size distribution of runs of homozygosity in each one of the three LWfG sampling groups. Error bars depict the standard deviation within groups. In all cases, pairwise comparisons are done using Tukey's HSD tests (NS: p-value > 0.05, *: p-value < 0.05, and **: p-value < 0.01).

Heterozygosity estimates indicated that all LWfG birds have overall high levels of genomic diversity respect the other goose species (Supplementary Information 2.2.2, Figure S9). When focusing on LWfG alone, we found that the Swedish birds display slightly lower genomic diversity than both Russian and Norwegian LWfG birds (Fig. 4A). These results were also confirmed when analyzing the dataset mapped to the mallard duck (Figure S9).

Finally, we estimated an average inbreeding level of 0.052 for all LWfG (5.2% of their genome allocated in runs of heterozygosity), with significantly higher inbreeding levels in the Swedish individuals (F_ROH_ = 0.082) than in either the Russian (F_ROH_ = 0.01) or the Norwegian (F_ROH_ = 0.009) individuals (Fig. 4B). In fact, most Swedish birds were identified as close relatives of different degrees (Supplementary Information 2.1.2). Additionally, we identified that the increased inbreeding in the Swedish birds can be attributed to an excess of both short (< 2 Mb) and long (> 2 Mb) ROHs (Fig. 4C, Figure S12). As in the rest of the analyses, the replicated tests performed on the data mapped to the mallard confirmed the above inbreeding results (Supplementary Information 2.2.2).

## Discussion

We found no evidence, in either the nuclear or mitochondrial genomes, suggesting an excess of introgressed alleles from GWfG, or any other goose species, into the wild Swedish LWfG population relative to the Norwegian or Russian ones. Given our sample size and the resulting low probability of non-detection, we therefore consider it highly unlikely that the reinforcement programme between 1981 and 1999 led to the introduction of introgressed genes into the wild Swedish population.

We note that there are several competing hypotheses that can generate a D-statistic value of zero (no introgression) in our nuclear genomic comparisons. First, it is possible that there has been no introgression from the GWfG, or any other goose species, into either the Swedish, Norwegian or Russian LWfG populations. A second possibility is that there has been introgression from GWfG into the LWfG, but that this happened into the ancestral lineage of all extant LWfG populations. This would have resulted in all populations carrying the same amount of GWfG ancestry, and thus D-statistic values of zero in all comparisons. This second scenario would be consistent with the observations by Ottenburghs et al.^2,4^, which suggested that introgression during the early stages of speciation has been a common feature among all goose species including the LWfG. A third scenario that could explain our results would be that there have been multiple separate introgressions into all three populations, Sweden, Norway and Russia. Although theoretically possible, we consider this scenario extremely unlikely since the level of introgression into the separate populations must then have been more or less exactly the same in order to result in D-statistic values of zero in all comparisons.

Our mitochondrial analyses also suggested lack of GWfG introgression into LWfG. Although our results grouped GWfG and LWfG into reciprocally monophyletic clades, we note that precise mitochondrial reconstructions were impeded by the presence of seemingly widespread mitochondrial insertions in the nuclear genome (numts). Numts are known to be present in bird species such as ducks and geese^28^. In fact, one of the first characterizations of a numt in birds was done in snow geese^29^. Even though the existence of numts has been highlighted in previous mitochondrial-based studies on LWfG^17^, our results suggest that they could be much more prevalent than previously estimated, possibly spanning the entire mitochondrial sequence. Further analyses, for example making use of long-read DNA sequencing technologies, will be needed to better identify and characterize these numts.

Not finding GWfG introgression into the Swedish LWfG population is contrary to the expectations based on previous studies, which had suggested that GWfG alleles were introgressed in 16% of captive-bred LWfG individuals^1,6^. Because these samples included birds from the breeding programme at Öster Malma that were used to reinforce the Swedish wild population between 1981 and 1999, it has been assumed that GWfG alleles were present in the current wild Swedish LWfG population^8^. However, exactly what proportion of the released LWfG birds actually carried GWfG alleles, and thus acted as vectors to the wild population, was unknown.

We caution that we cannot fully exclude the possibility that some introgression did occur during the release programme between 1981 and 1999, where hybrid birds were at such a selective disadvantage that they left no detectable signature in the population 3–5 generations later. However, we consider it highly unlikely that such a selective disadvantage would have removed also neutral SNPs to the extent that we could not detect any signal in the D-statistic tests, unless first generation hybrids between resident and released birds effectively had zero fitness. Supplementing or translocation projects using stocks that have spent several generations in captivity may indeed show lower success compared to breeding programmes based on wild-caught individuals (e.g. Robert^30^). In captivity, relaxation from natural selection can affect genetic characteristics as well as learned behaviours^31^. Consequently, viability, survival and the ability to recruit individuals into existing wild populations decrease with the duration of a reinforcement project^32,33^. The LWfG released in Sweden in the period 1981–1999, which originated from a stock heavily influenced by captive park birds and collections, probably had low ability to adjust to the wild. Hence, instead of selection against hybrids, we hypothesize that few or no released birds that carried introgressed GWfG genes may have contributed to the Swedish population’s present-day genetic variation, either due to random chance or because they did not adjust to the wild. Finally, it is also possible that the management decision of halting the releases as soon as hybrid birds were detected among the captive-bred LWfG successfully prevented the introduction of any founders with GWfG ancestry into the wild population, which may have eventually occurred had the releases continued.

In addition to no evidence of introgression, we also found that the Swedish population is genetically distinct from both the Russian and Norwegian populations (*p* < 0.001; F_ST_ = 0.069 and 0.068, respectively, Table S3). By comparison, this level of divergence is on par with F_ST_ values reported between West African and European human populations^3,4^. There could be several different explanations to the distinctiveness of the Swedish LWfG population. For example, it might reflect a different postglacial origin of the Swedish population compared to those in Russia and Norway, a common feature in Anatidae species^35^. Alternatively, the high divergence could be a consequence of strong genetic drift in the Swedish population during the last 100 years of abrupt population decline. Finally, by incorporating individuals from parks in the Netherlands and England, the release programme between 1981 and 1999 may have resulted in a significant shift in genetic composition of the wild Swedish population. Resolving these questions would be highly interesting but will require a much wider spatial and temporal sampling effort.

In contrast to the high differentiation of the Swedish population, we found that the Norwegian and Russian LWfG birds are genetically indistinguishable (F_ST_ = 0, *p* = 0.471, Table S3). These results suggest that even though the Norwegian (Fennoscandian) and Russian (Western Main) populations have different migratory routes (Fig. 1), they have in fact a very close relationship, probably explained by occasionally shared wintering and breeding grounds^36,37^ and the less philopatric males pair-bonding and mating with females from other subpopulations, thereby reducing subpopulation differentiation in the nuclear DNA. The lack of differentiation between the Norwegian and Russian samples is consistent with the results from an earlier genetic study on museum specimens^3,8^, which identified a temporal increase in genetic diversity in the Norwegian LWfG population from historical times to present day, and suggested this has been caused by an increased male-mediated genetic influx from the Russian populations in the last decades. Interestingly, our results suggest that this increased gene flow does not seem to have affected the Swedish reinforced population.

Our results also showed that the Swedish population had significantly lower genome-wide diversity and higher inbreeding levels compared to the Russian and Norwegian populations. It should be noted here that the LWfG as a species has one of the highest levels of genetic diversity among birds^39,40^. Nonetheless, the comparatively lower diversity in the Swedish population signifies a reduced potential to adapt to changes in the environment^41^. Also, the increased amount of inbreeding in the Swedish population, and in particular the excess of long RoHs observed in our analyses, suggest that the Swedish population has been subject to recent mating between close relatives^42,43^, which may be explained by a generally small population size and/or significant influence from the captive breeding programme.

Lower genetic diversity and higher levels of inbreeding could have suggested that the present-day Swedish population is subject to genetic threats in the form of inbreeding depression and increased genetic load. However, around the time that our Swedish samples were collected, a second reinforcement programme was implemented in Sweden. This second programme has successfully been releasing birds (an average of 46 individuals per year) that originate from the Russian population^4,4^. While originally aimed to dilute and reduce the impact of putative GWfG introgression, this second release programme may have resulted in a restoration of genetic diversity and mitigation of inbreeding in the Swedish population, thus mimicking the effect of recent natural gene flow from Russia into Norway^3,8^. Since the first evidence of successful reproduction of a released bird with Russian origin was in 2016, and all the released birds are individually ringed, it is certain that none of the Swedish samples analyzed in this study (including the two birds sampled in 2015 and 2016) had any ancestry from Russian birds released during this second reinforcement. Therefore, future studies on samples collected more recently will be needed to monitor the consequences of the current reinforcement programme on the distinctiveness, genetic diversity and inbreeding levels in today's Swedish LWfG population.

## Conclusions

Our findings showcase the risk of basing management decisions in translocation and reinforcement projects solely on data from captive breeding stock individuals without carefully monitoring the wild populations. After being released into the wild, captive-bred animals face a set of new challenges, such as severe predation rates^4,5^, which critically determine the ability of the newly introduced animals to contribute to the wild recipient population’s gene pool. Conservation legislation and policies, such as the Habitats Directive of the European Union, often mandate the restoration of populations that are extinct or close to extinction, for example through reinforcement^4,6^. Low levels of gene flow, even from a divergent population, can provide a substantial demographic and genetic boost to small populations, which may allow them to recover and withstand environmental and genetic stochasticity^47,48^. Despite this, conservation biologists have generally tended to shy away from routinely crossing populations^4,9^. One of the reasons for this, as exemplified by the Swedish LWfG population, is the fear of outbreeding depression and/or genomic contamination through hybridization, which may lead to reduced population viability. In fact, with a growing anthropogenic footprint, there is an increased risk of such human-mediated genomic contamination^2,50,51^. However, as shown in this study, population genomics offers a highly powerful way to assess the levels of hybridization in threatened populations. A genomic approach should also make it possible to manage the risk of introducing hybrids into the wild through genetic testing of captive individuals before releasing them. In addition, genomics can also be used to carefully monitor the wild population during and after such releases. We think this should be the norm, not the exception, whenever these kinds of projects are planned.

## Methods

### Samples and dataset

DNA samples used in this study were collected from blood extracts of live birds during conservation efforts. Sampling permits were issued by the Swedish Board of Agriculture (Jordbruksverket), Nordens Ark and the Swedish Association for Hunting and Wildlife Management (Jägareförbundet), and were approved by the ethical boards Uppsala Djurförsöksetiska Nämnd and Göteborgs Djurförsöksetiska Nämnd with permit numbers C171/10, 257/2011, and 140/2014. All sampling was carried out in accordance with the relevant guidelines and regulations.

We collected blood samples from 21 LWfG birds from three different populations: 12 at known stop over sites of the Swedish population (Figure S1); 5 from Russian breeding grounds at the Nentsien Autonom Okrug; and 4 from Norway from local nesting birds (Table1, Supplementary Information 1.1). The blood samples from the Norwegian birds were obtained from the existing collections at BirdLife Norway (collection IDs VA7, VA8, VA12, and VA14, Table 1). Genomic DNA was extracted using a KingFisher Cell and Tissue DNA Kit (Thermo Fisher Scientific, MA, USA) and genomic libraries for whole genome sequencing built using the TruSeq PCR-Free protocol (Illumina, CA, USA). Genomic libraries were then sequenced on five Illumina HiSeqX lanes using 2 × 150 bp paired-end read settings.

Raw reads were mapped against the pink-footed goose genome^52^ using the *BWA mem* algorithm^53^ with default parameters (Table 1, Supplementary Information 1.3). In order to detect possible biases in some analyses, we also mapped the raw reads to the mallard duck (*Anas platyrhynchos*, CAU_duck_1.0^54^) genome, which has a better quality assembly but is much more distantly related. The publicly available raw data from 22 other goose samples from 18 different species^24,25^ and the raw reads from the PfG sample that was used to generate the de novo assembly^52^ were mapped to both references using the same settings (Table S1). Thus, the final datasets contained 42 genomes from 18 different goose species (Table 1, Table S1).

### Variant discovery

We identified variants in each sample using *bcftools mpileup* and *bcftools call*^5,5^ and filtered those calls by keeping only biallelic variants on positions covered at least seven times (i.e. minimum read depth 7), they are present in more than 90% of the samples, and with genotype quality > 30 (Supplementary Information 1.4). Heterozygote variants were only kept if they had an allelic balance > 0.2 and < 0.8. We then used RepeatMasker^5,6^ and RepeatModeler^5,7^ to identify repetitive regions in both reference genomes, and excluded all variants allocated on those regions as well.

### Computational analyses

We used the normalized ratio of reads mapping to chromosome Z and the ones mapping to an autosome of similar size (chromosome 4) to determine the sex of each newly sequenced LWfG bird^58^ (Supplementary Information 2.1.1). Kinship within the LWfG samples was investigated using the tool *–relatedness2* from vcftools^59^ (Supplementary Information 2.1.2).

We used principal component analysis (PCA) to explore the broad genetic affinities among the LWfG, GWfG and PfG samples in the dataset, and also within the LWfG samples alone (Supplementary Information 2.1.3). Genomic differentiation (F_ST_) between the three LWfG populations was estimated using the tool *-weir-fst-pop* in vcftools, which estimates differentiation values between groups of samples using a weighted F_ST_^60^, and assessed significance using a permutation test (Supplementary Information 2.1.4). Genetic affinities and possible admixture events among GWfG, PfG and LWfG were further explored using the maximum likelihood approach of *TreeMix*^61^ (Supplementary Information 2.1.5).

We estimated per sample genome-wide heterozygosity directly counting heterozygote and homozygote genotype calls (‘hard-calls’) from the filtered VCF files (Supplementary Information 2.2.1). Long runs of homozygosity (> 100 kb), stretches of the genome with none or very limited number of heterozygote sites, were identified in each sample using the *–homozyg* option in Plink^62^ (see Supplementary Information 2.2.2 for details on settings and thresholds), and used to estimate per individual inbreeding coefficients (F_ROH_).

In order to identify introgression from other goose species into the Swedish LWfG birds we estimated D-statistics, also known as ABBA-BABA tests^19^ (Supplementary Information 2.3.1), in popstats^63^. We first investigated GWfG introgression in Swedish LWfG samples respect to the Norwegian or Russian ones by performing tests of the form D(SWE, RUS/NOR; GWfG, PfG), where SWE are all possible Swedish LWfG birds, RUS and NOR are all possible Russian and Norwegian birds respectively, the GWfG sample is used as donor, and the pink-footed goose as outgroup. We then checked for possible reference biases by performing the same tests using the bar-headed goose (*Anser indicus*, BhG) as outgroup (i.e. D(SWE, RUS/NOR, GWfG, BhG)), and repeated all tests using the dataset mapped to the mallard duck (Supplementary Information 2.3.1). Additionally, we explored evidence of introgression from all the other *Anser* species in the dataset into the Swedish LWfG population using tests of the form D(SWE, RUS/NOR; X, BhG), where X is any of the possible *Anser* species tested as donors, and the bar-headed goose as outgroup. We used the same settings as above for all tests and replicated them using both the data mapped to the PfG assembly and to the mallard duck assembly (Supplementary Information 2.3.1).

Finally, we reconstructed the mitochondrial genomes of our LWfG samples from the shotgun sequencing data using the mitogenome of a GWfG as reference (NC_004539.1). We then built a maximum-likelihood tree of the LWfG mitogenomes together with the previously published mitogenomes of two bar-headed geese and four GWfG (Supplementary Information 2.3.3).

## Supplementary information

Supplementary Information

## Data Availability

All the raw data generated for this manuscript are available at the European Nucleotide Archive (ENA), accession number PRJEB40857.
