# Supplementary material for: Population genomics reveals lack of greater white-fronted introgression into the Swedish lesser white-fronted goose
Source: Sci Rep. 2020 Oct 27;10:18347. doi: 10.1038/s41598-020-75315-y (PMC7591532; doi:10.1038/s41598-020-75315-y)
Supplement: Supplementary file 1 — Supplementary Information [file 41598_2020_75315_MOESM1_ESM.docx]

**Supplementary Information**

**Population genomics reveals lack of greater white-fronted introgression into the Swedish lesser white-fronted goose**

David Díez-del-Molino, Johanna von Seth, Niclas Gyllenstrand, Fredrik Widemo, Niklas Liljebäck, Mikael Svensson, Per Sjögren-Gulve, Love Dalén

**Overview**

**1. Sampling and data processing**

**1.1 Samples**

**1.2 DNA extraction and sequencing**

**1.3 Data processing and final datasets**

**1.4 Repetitive regions and variant discovery**

**2. Data analyses**

**2.1 Population structure and differentiation**

2.1.1 Sex determination

2.1.2 Kinship

2.1.3 Principal Component Analysis

2.1.4 Genomic differentiation

2.1.5 TreeMix

**2.2 Genomic diversity and inbreeding**

2.2.1 Genome-wide heterozygosity

2.2.1 Inbreeding

**2.3 Introgression**

2.3.1 D-statistics

2.3.2 Detection probability

2.3.3 Mitochondrial analyses

**3 Supplementary references**

**1. Sampling and data processing**

**1.1 Samples**

1.1.1 Swedish samples
All the samples representing the Swedish population of lesser white-fronted goose (LWfG) were taken from birds that had been ringed when visited the Hudiksvall during moult or spring migration. Birds were given individual colour rings (leg). Four of them were also tagged with tracking devices (transmitters or data loggers), so the movement of these birds could be followed closely.

Our data shows that they regularly visit known key sites for the Swedish population (staging, breeding and moulting areas), indicating that they are representative of the population (Figure S1).


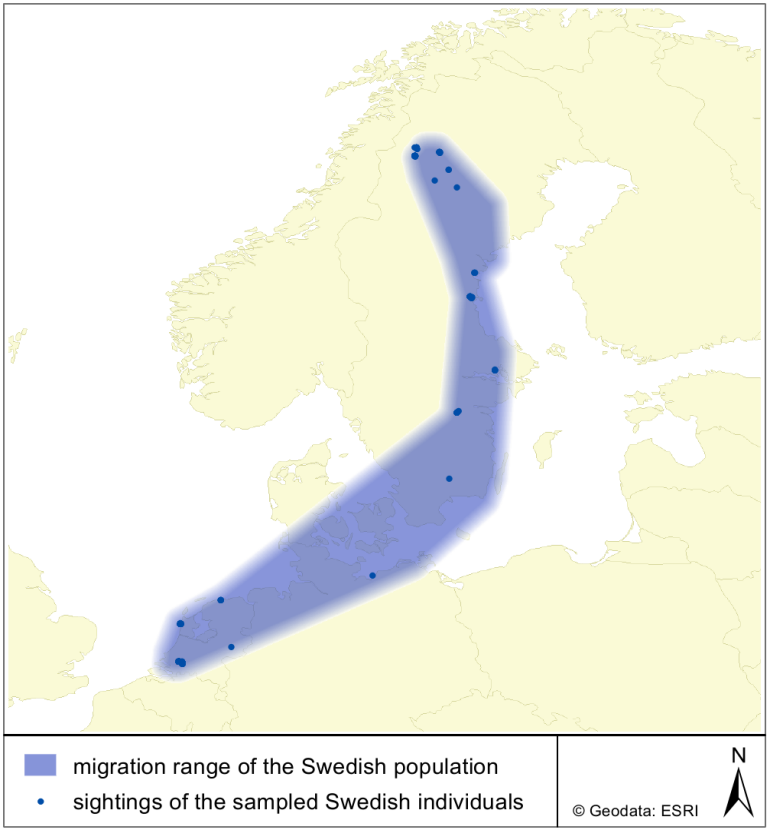


Figure S1. Schematic overview of flyways used by Swedish lesser white-fronted goose. Data from Swedish birds depict the boundaries of the area where sightings of marked birds were done, including tracking data from 4 individuals with satellite transmitters (unpublished data from Projekt Fjällgås, jagareforbundet.se/en/projektfjallgas). Resightings of sampled birds from Swedish population in this study as dark blue points (n=611). Map was created with ESRI’s ArcMap v10.

*2010 campaign*
Samples from the year 2010 (July) were collected on a ringing effort during moulting. A total 19 lesser white-fronted goose were ringed out of a flock of 31 birds, 10 of them were sampled for DNA. In 2010, the estimated census size was 110 birds. All ringed birds were aged but not sexed.

2*015 campaign*
Three LWfG were sampled during spring migration at the stop over site in Hudiksvall. The aim of this campaign was to put satellite transmitters to the birds in order to precisely map the movements of the wild Swedish population, including site use and migration routes. One of the individuals (male) was a re-capture from the 2010 campaign. Samples used here are from adult male that was equipped with a tracking device. This male was at least three years old when sampled. In 2015, the census size was estimated to be only 45 birds.

*2016 campaign*
In 2016 an adult female was sampled while staging in Hudiksvall. This female was paired with an unringed male, and it was at least three years old. In autumn of 2016, the estimated census size was 56 individuals.

1.1.2 Russian samples
Our four Russian samples come from birds captured as goslings (3-5 weeks of age) in the breeding grounds of the Nentsien Autonom Okrug. These were later transported to the Moscow Zoo and subsequently exported to Sweden to become founders of the captive breeding population (2010 - today). During the years 2005-2013 a total of 59 birds were exported this way from Russia to Sweden and contributed to this breeding program. To avoid getting many siblings, a maximum of two goslings per family were captured.

1.1.3 Norwegian samples

DNA samples from Norwegian LWfG were obtained from the existing collections at BirdLife Norway. All samples were collected in Finnmark in year 2000. One of the sampled birds (VA4) was sampled at a spring stopover site. This bird was seen last time in spring 2004 having produced chickens in years 2002 and 2003. The other three samples were collected from birds captured in autumn suggesting that these are local nesting birds.

**1.2 DNA extraction and sequencing**

Genomic DNA from blood from 21 samples was extracted using a KingFisher Cell and Tissue DNA Kit (Thermo Fisher Scientific, MA, USA) on an extraction robot following the manufacturer’s instructions. DNA was quantified on a Qubit instrument (Invitrogen, CA, USA) before sequencing. Genomic libraries and sequencing were done at SciLifeLab (NGI Stockholm). Libraries were prepared using a TruSeq PCR-Free protocol (Illumina, CA, USA) and sequencing was performed on five HiSeqX lanes using 2x150bp paired-end read settings.

**1.3 Data processing and final datasets**

Reads were mapped against the reference genome using the *BWA mem* algorithm [[1](https://paperpile.com/c/e1U4If/O8a18)] using default parameters. For comparative purposes, we mapped the data to both the closely related pink-footed goose (*Anser brachyrhynchus*, hereafter PfG; [[2](https://paperpile.com/c/e1U4If/wSk86)]) and the mallard duck (*Anas platyrhynchos*, CAU_duck_1.0; [[3](https://paperpile.com/c/e1U4If/MHgt4)]), which is more distantly related to the LWfG but has a better quality assembly. Raw sequencing data from 22 other goose samples, including 18 different *Anas* and *Branta* species from [[4](https://paperpile.com/c/e1U4If/MZtSP),5] were downloaded from ENA (PRJEB20373) and mapped the same way than our newly sequenced data. We additionally remapped the raw reads from the PfG sample that was used to generate the *de novo* alignment in [[2](https://paperpile.com/c/e1U4If/wSk86)]. Thus, our final dataset contained 42 genomes from 18 different goose species, 21 of which are newly sequenced (Table 1, Table S1).

Table S1. List of goose samples from [4,5] downloaded for this study and their average genomic coverage (x, after filtering by MQ >30) when mapped to PfG and the mallard duck.

| Sample | Species | Common name | PfG | Mallard Duck |
| --- | --- | --- | --- | --- |
| AnAl01U02 | *Anser albifrons* | Greater White-fronted Goose | 18.81 | 16.11 |
| AnAn01U02 | *Anser anser* | Greylag Goose | 15.9 | 13.68 |
| AnBr01U02 | *Anser brachyrhynchus* | Pink-footed Goose | 20.66 | 17.74 |
| AnCy01F02 | *Anser cygnoides* | Swan Goose | 20.8 | 17.8 |
| AnEr01U01 | *Anser erythropus* | Lesser White-fronted Goose | 20.34 | 17.38 |
| AnFa01U01 | *Anser fabalis* | Taiga Bean Goose | 20.41 | 14.96 |
| AnIn01U01 | *Anser indicus* | Bar-headed Goose | 14.81 | 11.79 |
| AnSe01U01 | *Anser serrirostris* | Tundra Bean Goose | 20.18 | 12.17 |
| BrBb01M02 | *Branta bernicla* | Dark-bellied Brent Goose | 12.61 | 10.77 |
| BrBh01F02 | *Branta bernicla hrota* | Pale-bellied Brent Goose | 12.67 | 10.84 |
| BrBn01M02 | *Branta bernicla nigricans* | Black Brent Goose | 12.52 | 10.54 |
| BrCa01U02 | *Branta canadensis* | Canada Goose | 12.1 | 10.36 |
| BrHu01U01 | *Branta hutchinsii* | Cackling Goose | 12.7 | 10.92 |
| BrLe01U04 | *Branta leucopsis* | Barnacle Goose | 12.83 | 11.02 |
| BrRu01F01 | *Branta ruficollis* | Red-breasted Goose | 12.44 | 10.69 |
| BrSc01M02 | *Branta sandvicensis* | Hawaiian Goose | 12.89 | 10.91 |
| ChCa01F02 | *Anser caerulescens* | Snow Goose - Female | 13.04 | 11.13 |
| ChCa01M01 | *Anser caerulescens* | Snow Goose - Male | 13.22 | 11.27 |
| ChCn01U01 | *Anser canagica* | Emperor Goose | 12.79 | 10.92 |
| ChRo01M01 | *Anser rossii* | Ross' Goose | 13.07 | 7.19 |

**1.4 Repetitive regions and variant discovery**

We first scanned both reference genomes for repetitive regions that can induce spurious variant calls due to missalignments using RepeatMasker [6] and created a bed file with those regions that exclude repetitive tandems using RepeatModeler [7].

We found that 7.29% of the PfG assembly was composed of highly repetitive regions, with the majority of them allocated to long interspersed nuclear elements (LINEs, 4.16%, Table S2). A very similar amount of repetitive regions was identified for the mallard duck assembly (7.39%, 4.27% in LINEs).

After excluding these repetitive regions we called variants in each sample independently using *bcftools mpileup* and *bcftools call* [8]. Next, we filtered these files by keeping only biallelic variants on positions covered at least seven times, with genotype quality > 30. To avoid spurious calls from sequencing or mismapping errors, we also excluded heterozygote positions for which the ratio of reads with the reference allele divided by depth was below 0.2 or above 0.8. We then merged these VCF files keeping only variants that segregate from the reference genome, that is, positions for which at least one of the 42 samples had one or two alleles different from the reference. Finally, we keep only positions that are genotyped in more than 90% of the individuals.

We identified a total of 29,023,687 variable sites across the whole dataset mapped to the PfG and 32,935,624 when using the dataset mapped to the mallard duck.

Table S2. RepeatMasker statistics for the PfG and mallard duck assemblies.

|  | PfG | Mallard Duck |
| --- | --- | --- |
| Genome size (Gb) | 1.12 | 1.07 |
| SINEs | 563,822 | 195,928 |
| LINEs | 47,903,230 | 44,426,324 |
| LTRs | 10,904,842 | 10,389,873 |
| DNA elements | 1,089,239 | 763,258 |
| Unclassified | 3,374,721 | 2,639,563 |
| Small RNA | 125,868 | 62,774 |
| Satellites | 340 | 0 |
| Simple repeats | 14,439,018 | 16,985,795 |
| Low complexity | 3,030,379 | 3,294,376 |
| *Total masked (bp)* | 81,431,459 | 78,757,891 |
| *Fraction masked (%)* | 7.29 | 7.39 |

**2. Data analyses**

**2.1 Population structure and differentiation**

2.1.1 Sex determination

We used the approach in [9] to determine the sex of the 21 newly sequenced birds. For this analysis we only used the data mapped to the mallard duck, since the PfG assembly does not have chromosomal resolution. For each genome, we estimated the sex by counting the number of reads mapping to the chromosome Z relative to the number of reads mapping to an autosome of similar size (chromosome 4) normalizing both counts by their respective chromosome size. Since female birds have only one copy of chromosome Z, twice as many reads should map to their chromosome 4, and the ratio ChrZ/Chr4 should be close to 0.5. On the other hand, males have two copies of chromosome Z, and hence the ratio ChrZ/Chr4 should be around 1. Out of the newly sequenced birds we identified 11 as female and 10 as male (Figure S2).


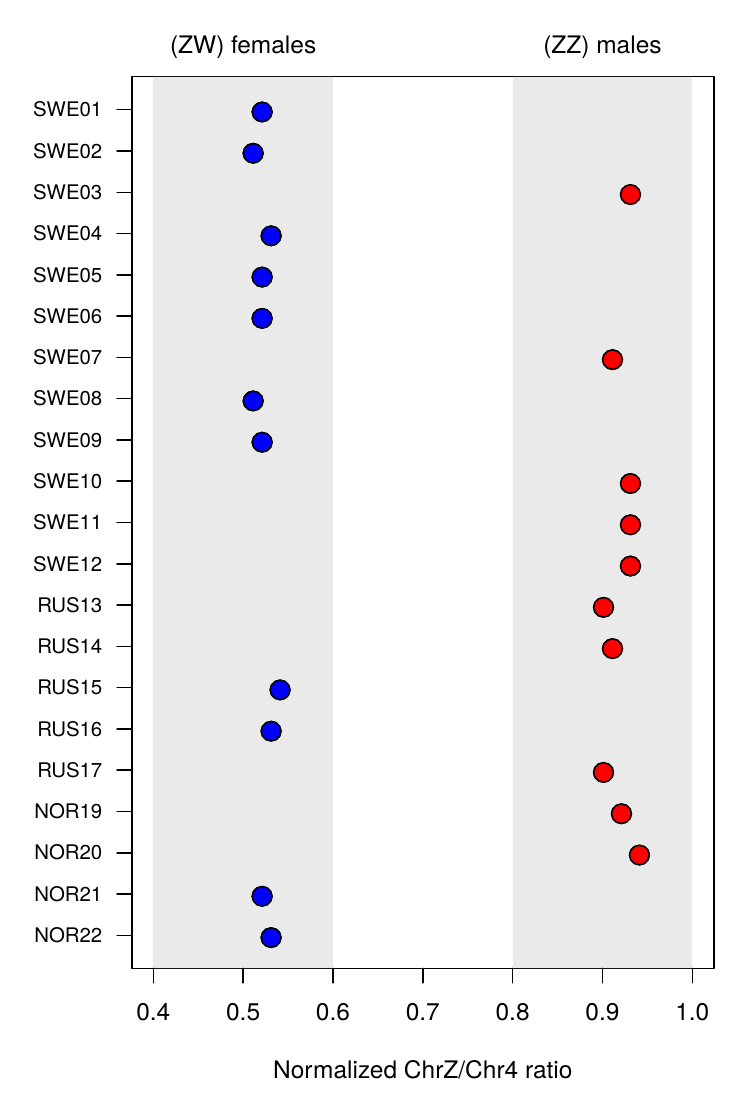


Figure S2. Sex determination. Normalized ratio of the number of reads mapping to a sex chromosome (ChrZ) versus an autosome (chromosome 4) of similar size in each sample. All samples are clearly identified as males (red dots, ZZ) or females (blue dots, ZW).

2.1.2 Kinship

We investigated relatedness among our LWfG samples using the tool *--relatedness2* from vcftools [10]. We used the datasets mapped to the PfG and to the mallard duck to estimate the pairwise kinship coefficient (Φ) for every pair of individuals and classify them as first, second and third degree relatives, or unrelated following [[1](https://paperpile.com/c/e1U4If/88z9A)1].

For both datasets, we found that most pairs of the Swedish LWfG analyzed have some degree of kinship between them, including two pairs clearly identified as first degree relatives (SWE1-SWE6 and SWE7-SWE10, Figure S3). Only one pair of the Russian (RUS13-RUS14) and none of the Norwegian birds sampled showed any degree of relatedness between them.


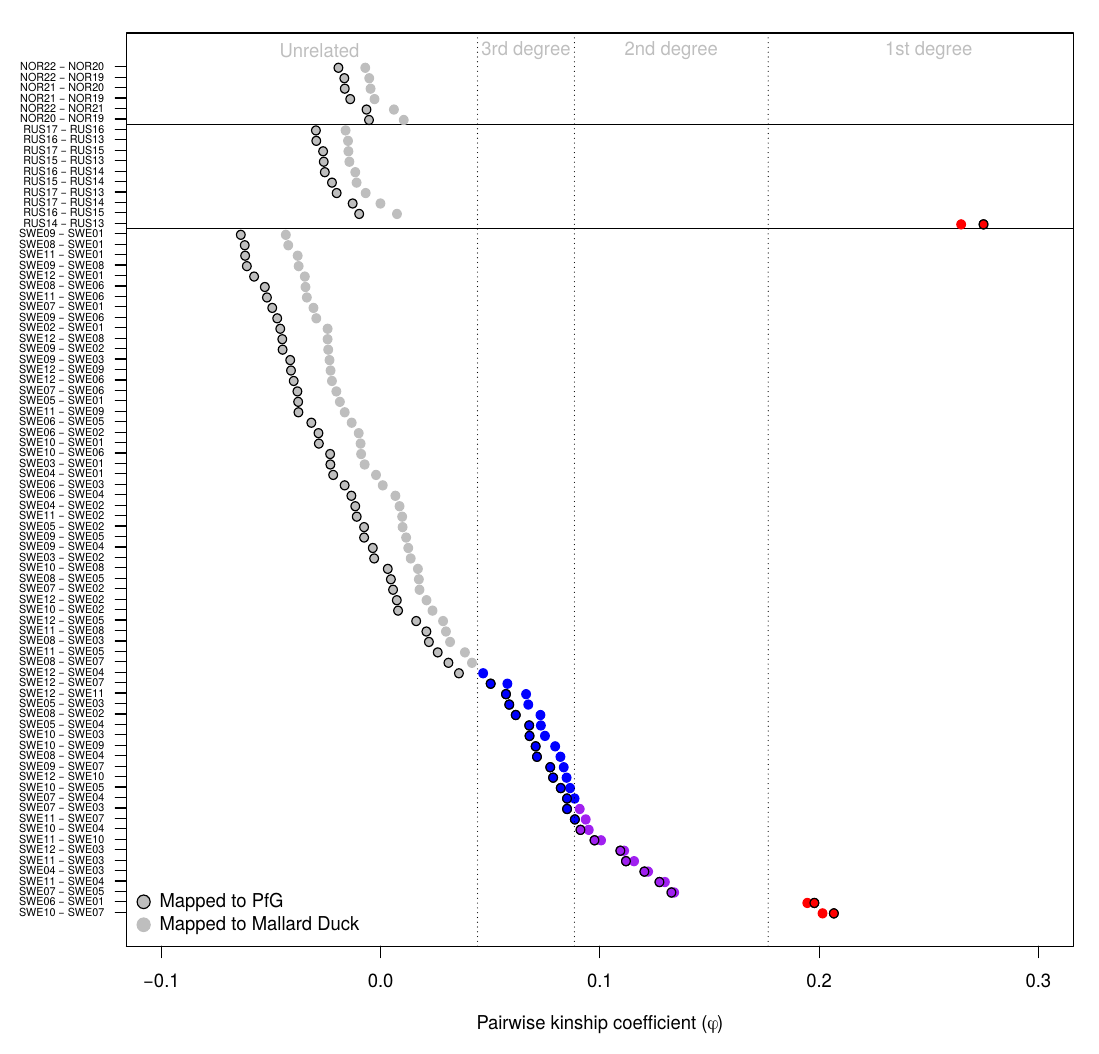


Figure S3. Pairwise kinship among all LWfG samples by population. Colored dots represent different degrees of kinship as defined in [[1](https://paperpile.com/c/e1U4If/88z9A)1]. Closed circles are the values for the dataset mapped to the PfG and open circles to the mallard duck.

2.1.3 Principal Component Analysis (PCA)

We explored the broad population relationships within all lesser white-fronted geese as well as those together with the greater white-fronted goose sample and the two PfG samples by summarizing their genetic variation onto the two main axes using principal component analysis (PCA). For each test, we first extracted the samples from the complete 42 sample VCF file keeping only positions variable between the samples to test *(--mac 1*), then pruned SNPs based on their linkage disequilibrium patterns (*--indep-pairwise 50 5 0.5*) and performed the PCA using Plink v1.9 *--pca* ([https://www.cog-genomics.org/plink2](https://www.coggenomics.org/plink2)). We repeated the same analyses for the dataset mapped to the PfG and the one mapped to the mallard duck.

The first two components of the PCA with three goose species segregate each bird in a distinct cluster for its species (Figure S4). In the PCA with LWfG samples only, the birds are structured in two discrete groups with the majority of Swedish samples in one of them and Norwegian and Russian samples in the other (Figure S5, upper left panel). Additionally, two Russian and two Swedish samples cluster together but differentiated from these two main groups. The LWfG bird from [[4](https://paperpile.com/c/e1U4If/MZtSP),5] seems to cluster with these two Swedish birds. The PCAs using the dataset mapped to the mallard duck produced the same general patterns (Figure S6). However, because these two distinct pairs of birds are among the birds found to be first degree relatives (see Supplementary Information 2.1.2) and this can interfere in the PCA, which assumes the independence of all samples, we repeated the analyses excluding one bird for each pair of relatives (SWE1, SWE7 and RUS13). In this new PCA, the first component clearly differentiates between Swedish and Russian/Norwegian birds, with SWE06 and the sample from [[4](https://paperpile.com/c/e1U4If/MZtSP),5] on an intermediate position (Figure S5D), whereas the second and third axes seem to relate exclusively to variation within Norwegian and Russian birds (Figures S5E, and S5F).


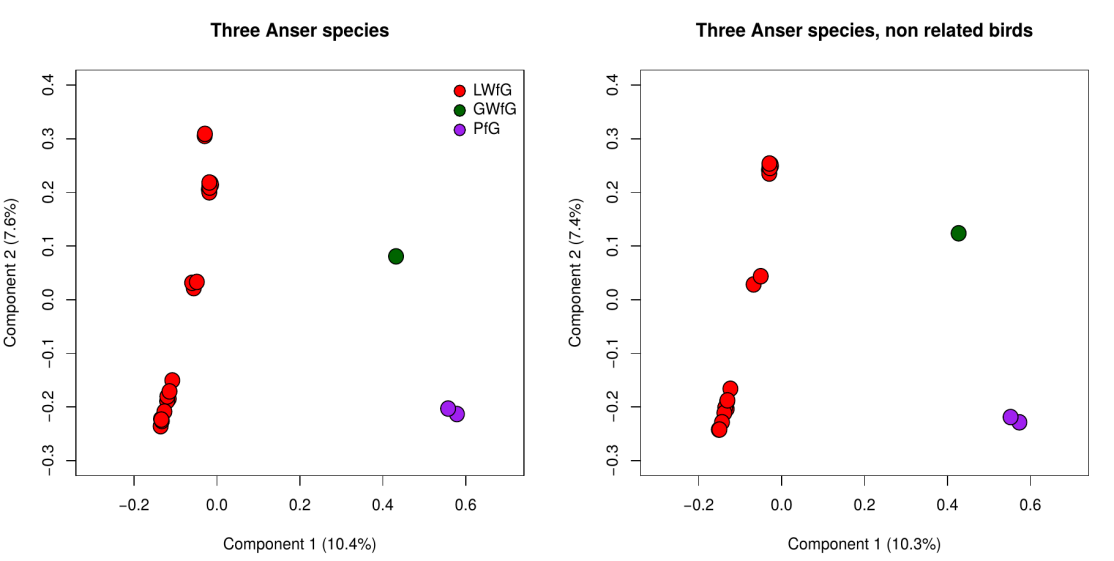


B

A

Figure S4. First two axes of a PCA of three goose species mapped to PfG. A) including all birds in the dataset. B) excluding SWE1, SWE7 and RUS13 to avoid first degree kinship relationships. Colors represent different species.


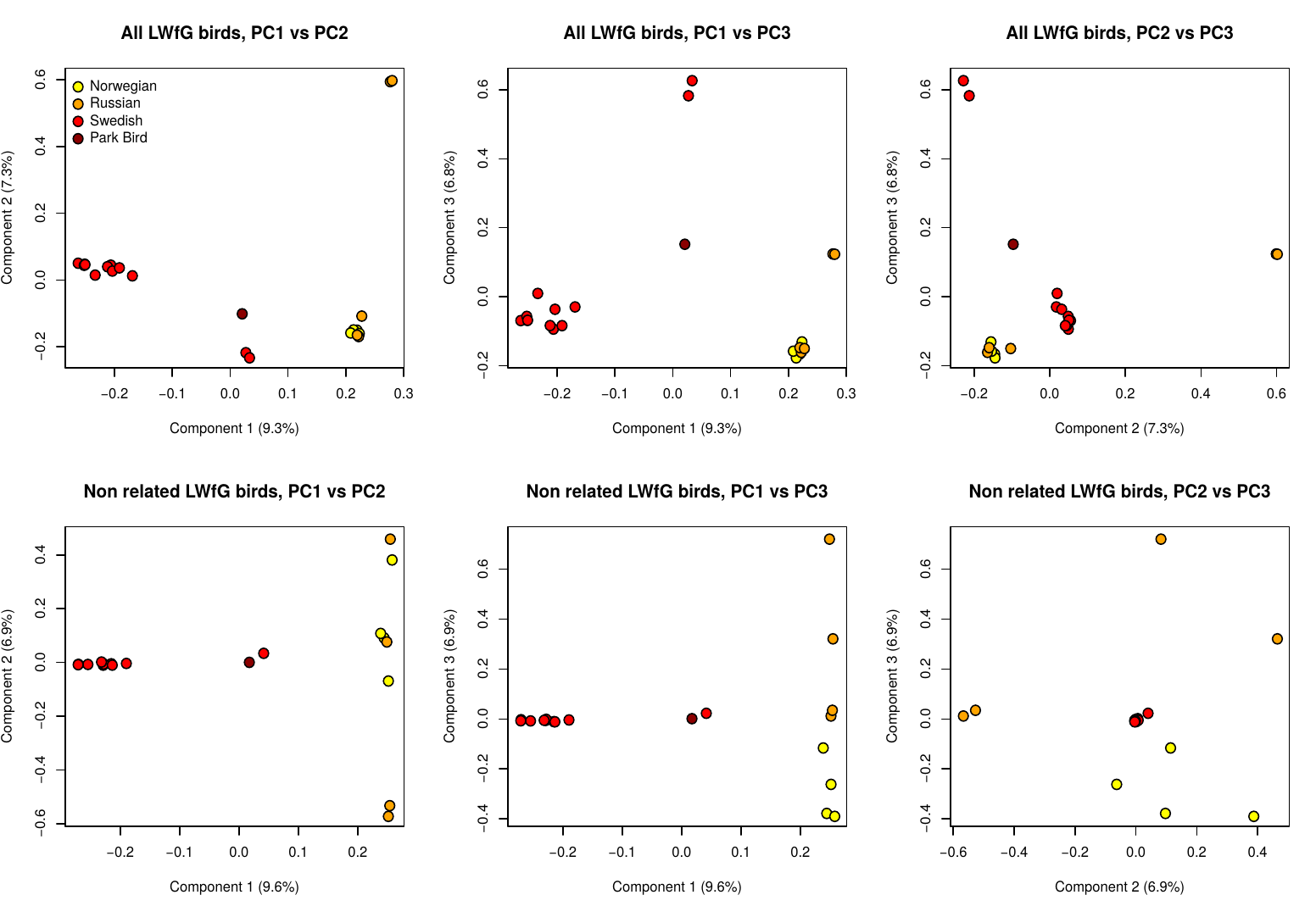


E

D

F

B

A

C

Figure S5. First three axes of the PCA with only LWfG birds mapped to PfG. A), B) and C), including all samples. D), E, and F) excluding the closely related samples.


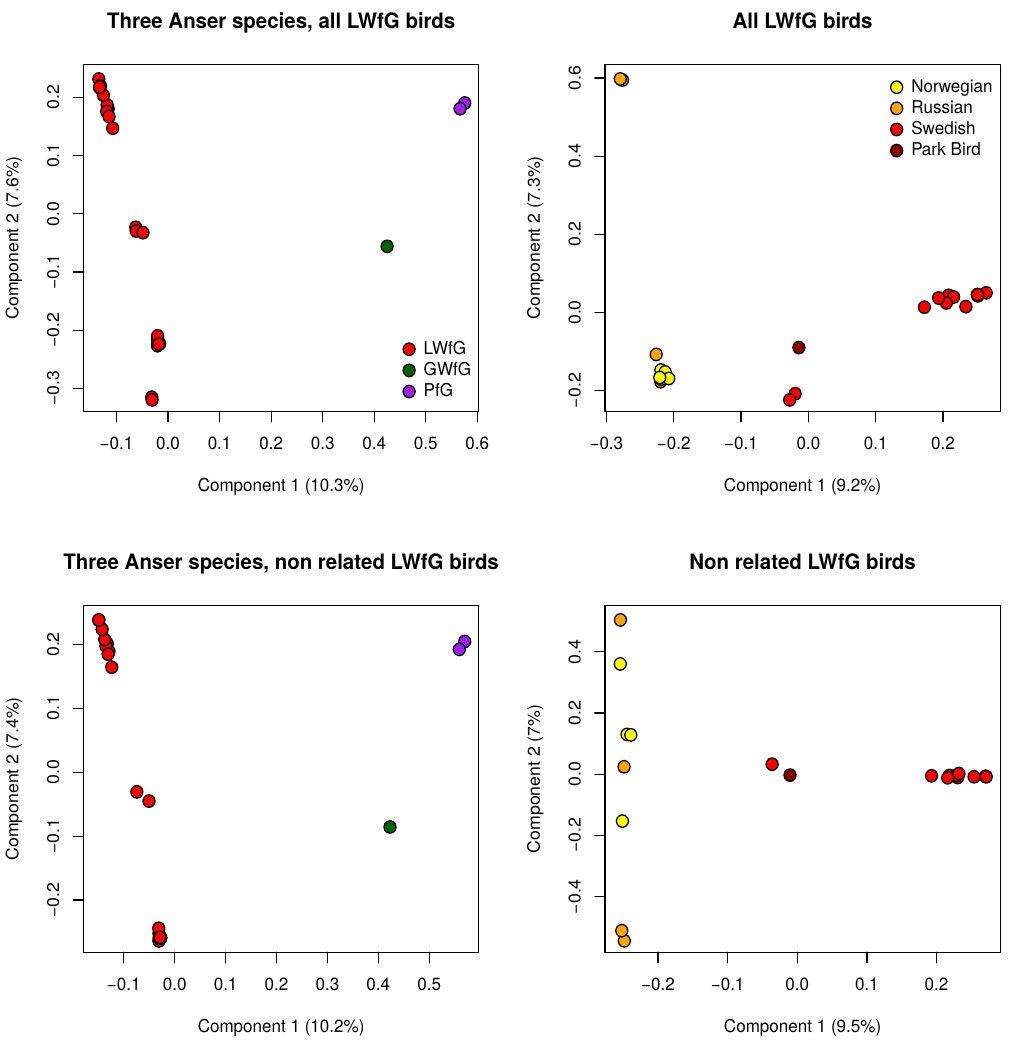


D

C

B

A

Figure S6. First two axes of a PCA of three goose species mapped to the mallard duck. A) and B) including all PfG, GWfG and LWfG birds in the dataset. Colors represent different species. C) and D) excluding the closely related LWfG birds. Colors indicate different populations of origin.

2.1.4 Genomic differentiation

We investigated pairwise differentiation levels between the three geographical groups of LWfG (SWE, RUS and NOR) using the *--weir-fst-pop* tool from vcftools, which estimates differentiation values between groups of samples using the weighted F_ST_ [[1](https://paperpile.com/c/e1U4If/CMtNA)2]. However, uneven or low sample numbers on some of the tested groups could lead to randomly positive F_ST_ values. Thus, to assess the significance of the obtained F_ST_ values and to test the robustness of the three geographical LWfG groups we implemented a permutation test. For each F_ST_ pairwise test, we performed 1,000 replicates on which we randomized the samples forming each group. We then estimated the significance as one-tailed P-value by calculating how many of the random replicas end up with an equal or higher F_ST_ than the observed one. In order to control for biases arising from the presence of close relatives, we performed all tests excluding three samples (see Supplementary Information 2.1.3).

Our results indicate significant differentiation between the Swedish birds and either the Norwegian and Russian ones, but not between the latter two populations (Table S3).

Table S3. Fst values between the three different populations in the LWfG dataset. Above diagonal: Weighted Fst [[1](https://paperpile.com/c/e1U4If/CMtNA)2]. Below diagonal: Permutation P-value (see text).

|  | SWE | NOR | RUS |
| --- | --- | --- | --- |
| SWE |  | 0.068 *** | 0.069 *** |
| NOR | *0* |  | 0.000 NS |
| RUS | *0* | *0.471* |  |

2.1.5 Treemix

We investigated the genetic affinities among LWfG birds and between them and the GWfG and PfG birds using *TreeMix* [[1](https://paperpile.com/c/e1U4If/GXhS6)3]. This method estimates the maximum likelihood tree for a set of samples using their allele-frequencies and a Gaussian approximation to genetic drift at the same time that fits a number of migration events between them, so it could also be informative of admixture events. We performed *TreeMix* analyses on all LWfG birds together with the GWfG and the two PfGs. The PfG bird used as reference was set as root for this analysis. Starting from the merged VCF file for all samples described above (see Supplementary Information 1.4), we first used *plink* to calculate allele frequencies using the flag *--freq* then converted the output into the *TreeMix* format using the script *plink2treemix.py*. *TreeMix* was run 10 replicates for each *m* value between 0 and 20 always using the -*-global* option and estimating standard errors in blocks of 5,000 SNPs. To summarize the results we chose the lowest *m* value that maximizes the gain of loglikelihood relative to the loglikelihood of the previous *m* value. Given that *TreeMix* analyses assume independence on the samples, we performed all analyses removing the three samples identified as first degree relatives (see Supplementary Information 2.1.3).

Among all models tested, the one with two migration edges (m=2) had the highest loglikelihood gain respect the previous value (Figure S7). The resulting tree indicated genetic affinities similar to the ones found in the PCAs, with the LWfG birds clearly forming two main groups, one with all the Swedish birds (including the park bird from [[4](https://paperpile.com/c/e1U4If/MZtSP),5]) and the other with the Norwegian and Russian ones (Figure 3C). In this model, the two migration edges identified suggested gene flow between Swedish samples (SWE8 to SWE11 and SWE2 to SWE8). The same general structure was evident also when using all other m values (Figure S8), and all migration edges up to 10 suggested gene flow within LWfG birds.


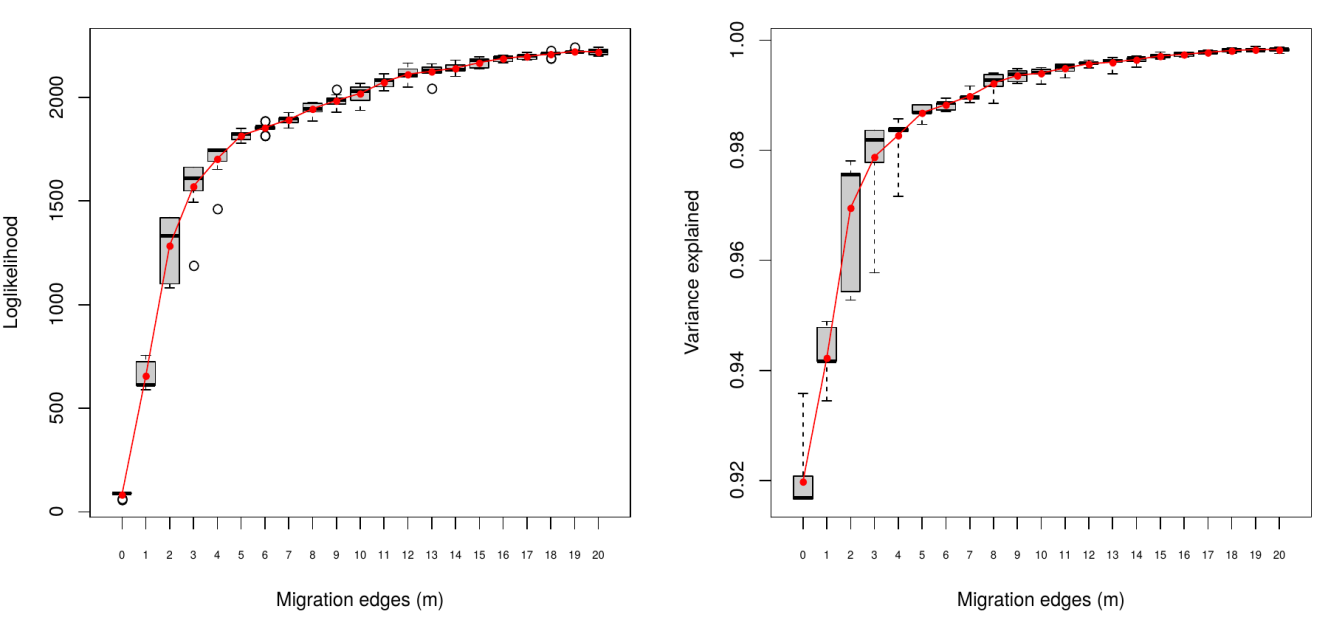


B

A

B

Figure S7. Loglikelihood and variance explained for all TreeMix models tested. Boxplots correspond to the values of the 10 replicates per *m*. Red dots represent the median values for each *m*. A) Loglikelihood per number of migration edges. B) Variance explained per number of migration edges.


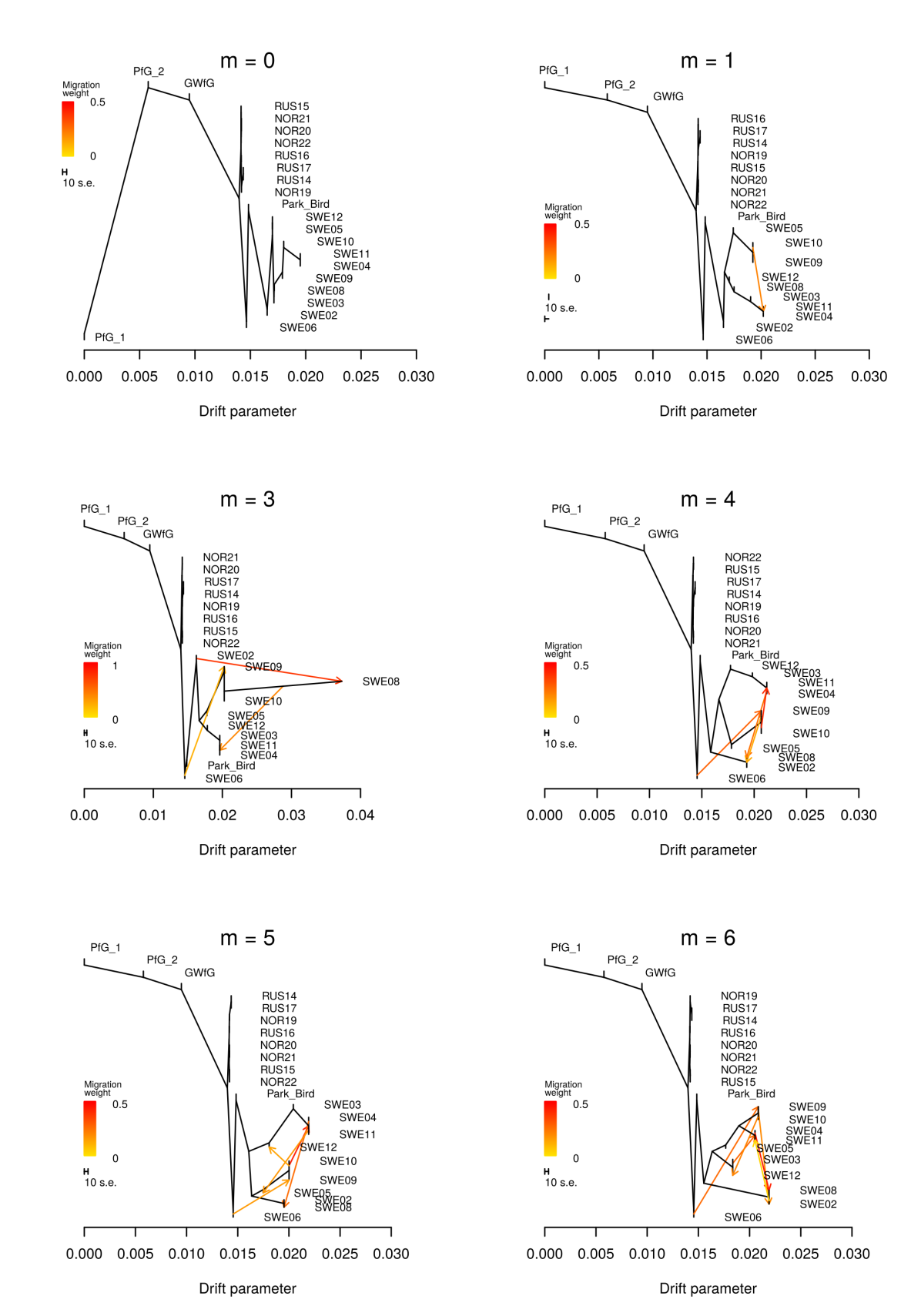


Figure S8. TreeMix reconstructed trees for m values 0 to 10 (m = 2 in main text). All trees are rooted on the sample PfG_1. Migration edges are depicted in a color scale depending on the inferred weight.


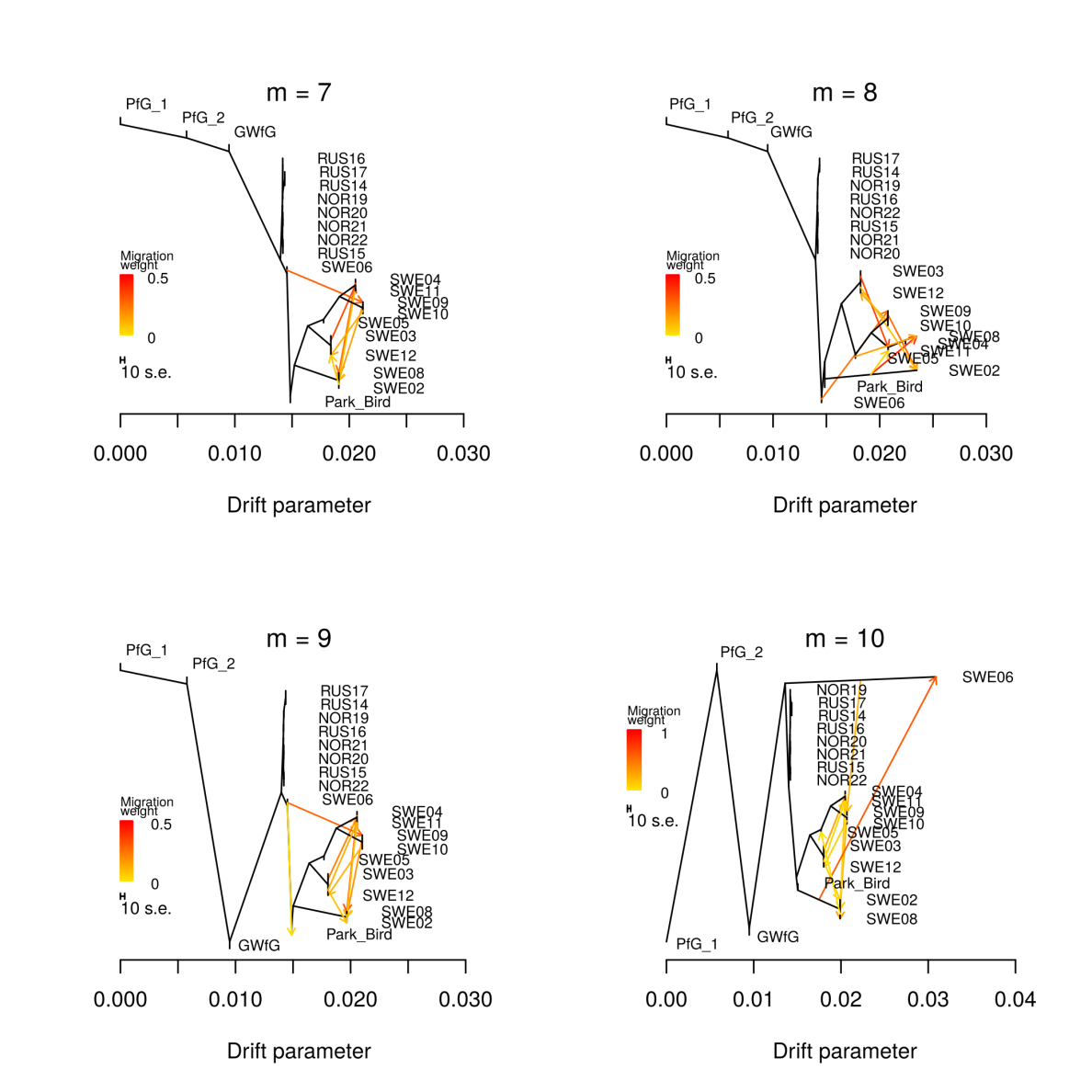


Figure S8 (cont).

**2.2 Genomic diversity and inbreeding**

2.2.1 Genome-wide heterozygosity

To estimate the per sample genomic diversity we applied an approach that directly counts heterozygote and homozygote genotype hard calls from the filtered VCF files (Supplementary Information 1.4) in sliding windows of 50kbp with a step size of 20kbp. We performed this approach in both the dataset mapped to the PfG and to the mallard duck.

Our estimates reveal that the LWfG birds analyzed here display genomic diversity levels on par with the most diverse other goose species in the dataset (Figure S9). Overall, the Swedish LWfG birds displayed lower genomic diversity than both Russian and Norwegian birds (Figure 4).


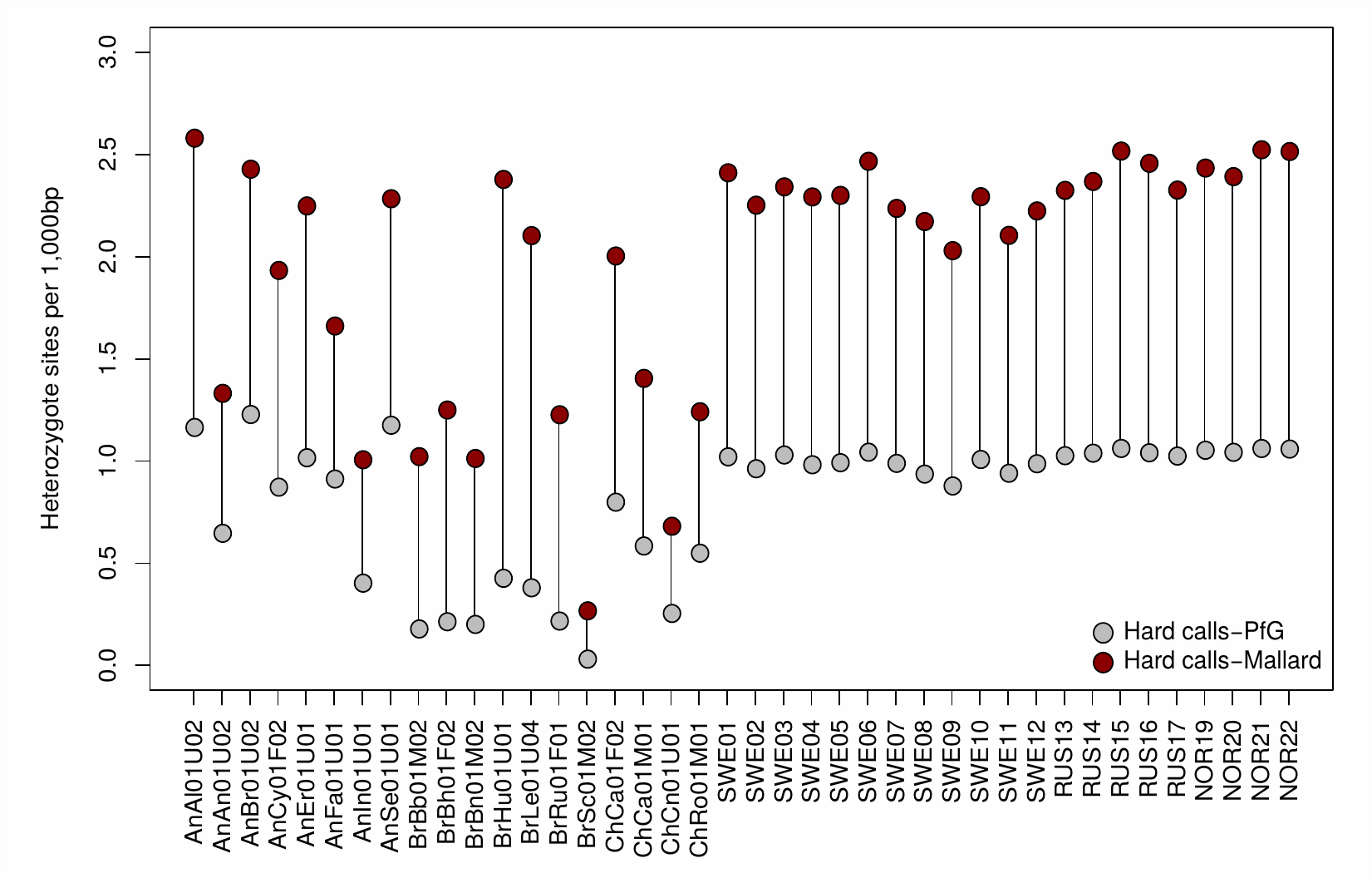


Figure S9. Comparison of estimates of genomic diversity for all samples mapped to the PfG and the mallard duck.

2.2.2 Inbreeding

To investigate inbreeding we estimated the per bird number, size and distribution of large stretches of genome sequence (>100kb) with none or very limited number of heterozygote sites, regions known as runs of homozygosity or ROHs [[1](https://paperpile.com/c/e1U4If/VEVhO)4]. Using the genotypes on the filtered and pruned plink files we estimated ROHs by using the sliding window approach of the *--homozyg* option of Plink v1.9 [[1](https://paperpile.com/c/e1U4If/Y3YKa)5] and the following settings: a minimum size of 100kbp (*--homozyg-kb* 100), a minimum of 25 SNPs per window (*-- homozyg-window-snp* 25), a threshold of 0.05 for overlapping homozygote windows (*--homozyg-window-threshold* 0.05), and a maximum of one heterozygote position within each window (*--homozyg-window-het* 1), a maximum of 1000 bp between neighbouring SNPs (*--homozyg-gap* 1000), a minimum SNP density of one SNP per 50 kb (*--homozyg-density* 50), a maximum of 5 missing sites per window (*--homozyg-window-missing* 5) and a high number of heterozygous sites within ROHs allowed (*--homozyg-het* 750) in order to prevent sequencing errors to cut ROHs.

When repeating all analyses using dataset mapped to the mallard duck assembly we observed that all samples showed much smaller ROHs, probably as an artifact due to the mallard duck being much more distantly related to the LWfG than the PfG. Therefore we increased the maximum of number of heterozygote positions within each window to 3 (*--homozyg-window-het* 3) to compensate for this. We then explored the inbreeding levels for each LWfG bird expressed as the total amount of genome allocated in ROHs (F_ROH_), as well as the size of the ROHs, which can be used to investigate the causes of inbreeding [[1](https://paperpile.com/c/e1U4If/VEVhO)4].

Our results indicate overall low levels of inbreeding for LWfG (average F_ROH_=0.052), but using both the datasets mapped to PfG and mallard duck we find a clear excess of inbreeding in Swedish LWfG birds with respect to both the Norwegian and Russian ones (Figure 4, Table S4). Swedish birds are also much more variable in their levels of inbreeding including birds from very low (i.e. SWE03, F_ROH_=0.012 ) to moderate (i.e. SWE09=0.191) inbreeding, and had generally longer ROHs including some larger than 20Mb (Figure 4, Figure S10, Figure S11). Furthermore, Swedish birds had an overall larger fraction of their genomes allocated in both long and short ROHs than the Russian and Norwegian ones (Figure S12).

Table S4. Inbreeding statistics for the LWfG samples and the two reference genomes.

|  | **PfG** | | | | **Mallard Duck** | | | |
| --- | --- | --- | --- | --- | --- | --- | --- | --- |
| **Sample** | **# ROHs** | **Average size (kb)** | **Total size (Mb)** | **F_ROH_** | **# ROHs** | **Average size (kb)** | **Total size (Mb)** | **F_ROH_** |
| SWE01 | 85 | 781 | 66.4 | 0.059 | 79 | 840 | 66.4 | 0.059 |
| SWE02 | 103 | 905 | 93.2 | 0.083 | 94 | 983 | 92.4 | 0.083 |
| SWE03 | 65 | 205 | 13.4 | 0.012 | 72 | 368 | 26.5 | 0.024 |
| SWE04 | 123 | 678 | 83.4 | 0.075 | 108 | 782 | 84.5 | 0.076 |
| SWE05 | 115 | 803 | 92.3 | 0.083 | 91 | 997 | 90.8 | 0.081 |
| SWE06 | 66 | 375 | 24.7 | 0.022 | 71 | 350 | 24.8 | 0.022 |
| SWE07 | 104 | 787 | 81.9 | 0.073 | 102 | 960 | 97.9 | 0.088 |
| SWE08 | 130 | 1,176 | 152.9 | 0.137 | 98 | 1,532 | 150.1 | 0.134 |
| SWE09 | 176 | 1,209 | 212.8 | 0.191 | 110 | 1,882 | 207.0 | 0.185 |
| SWE10 | 81 | 706 | 57.2 | 0.051 | 79 | 856 | 67.7 | 0.061 |
| SWE11 | 139 | 1,057 | 147.0 | 0.132 | 105 | 1,481 | 155.5 | 0.139 |
| SWE12 | 86 | 907 | 78.0 | 0.070 | 87 | 1,022 | 88.9 | 0.080 |
| RUS13 | 76 | 195 | 14.8 | 0.013 | 87 | 290 | 25.2 | 0.023 |
| RUS14 | 77 | 188 | 14.5 | 0.013 | 80 | 313 | 25.1 | 0.022 |
| RUS15 | 52 | 214 | 11.1 | 0.010 | 53 | 219 | 11.6 | 0.010 |
| RUS16 | 58 | 208 | 12.1 | 0.011 | 59 | 223 | 13.1 | 0.012 |
| RUS17 | 56 | 261 | 14.6 | 0.013 | 73 | 321 | 23.4 | 0.021 |
| NOR19 | 43 | 175 | 7.5 | 0.007 | 51 | 218 | 11.1 | 0.010 |
| NOR20 | 49 | 170 | 8.3 | 0.007 | 57 | 358 | 20.4 | 0.018 |
| NOR21 | 51 | 204 | 10.4 | 0.009 | 55 | 193 | 10.6 | 0.010 |
| NOR22 | 64 | 223 | 14.3 | 0.013 | 64 | 232 | 14.8 | 0.013 |


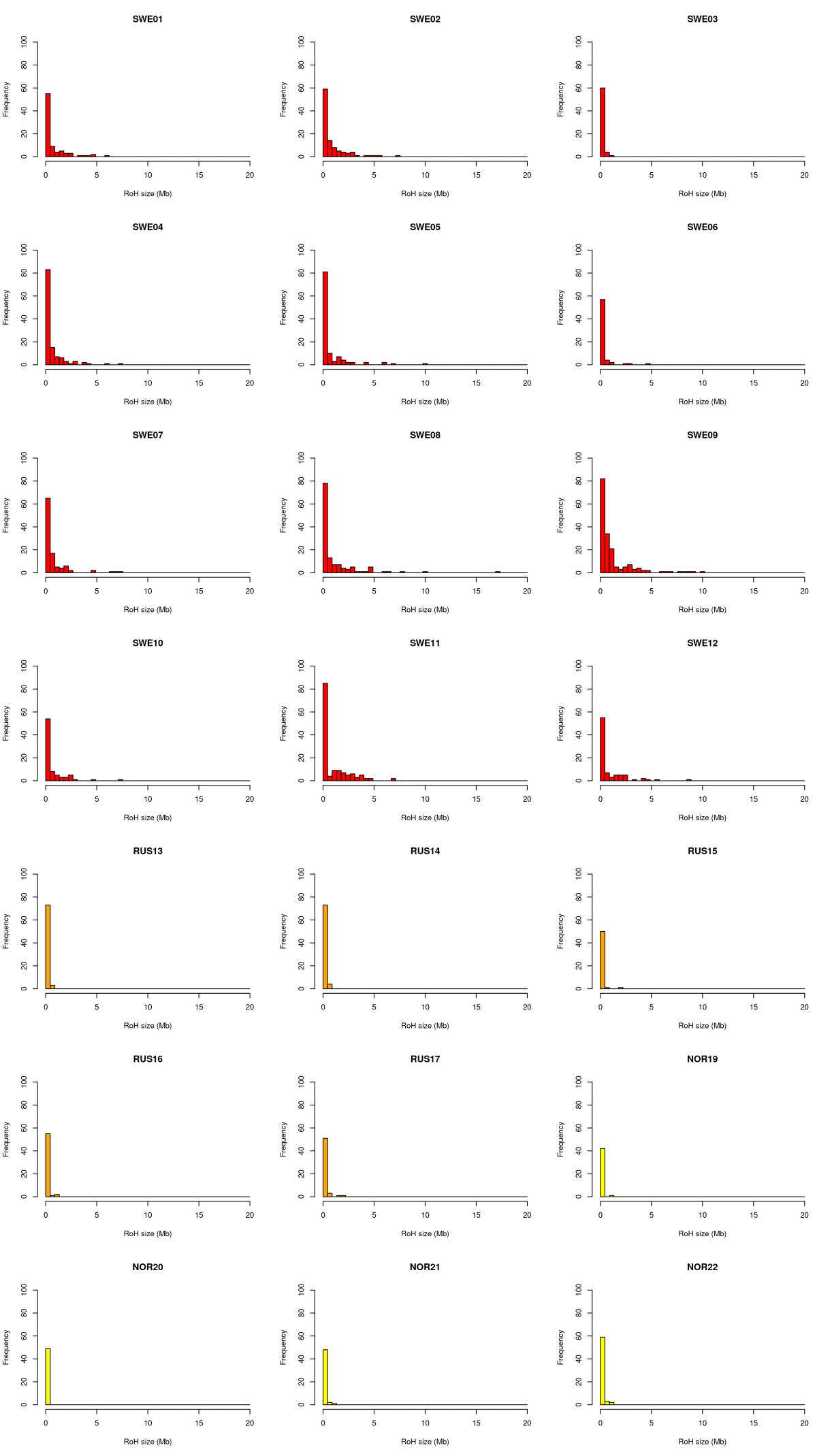


Figure S10. Size distribution of ROHs for each LWfG bird mapped to the PfG.


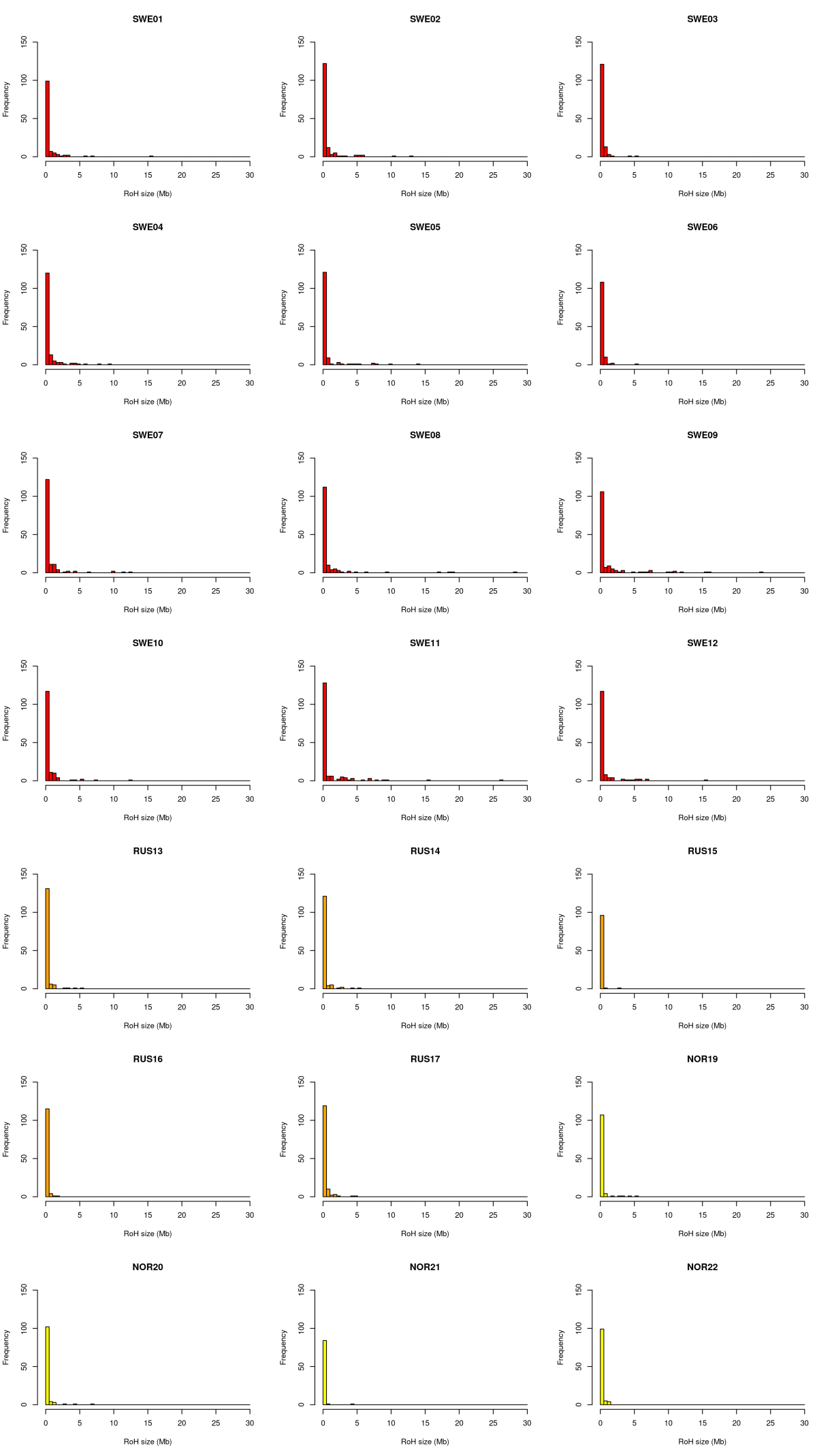


Figure S11. Size distribution of ROHs for each LWfG bird mapped to the mallard duck.

B

A


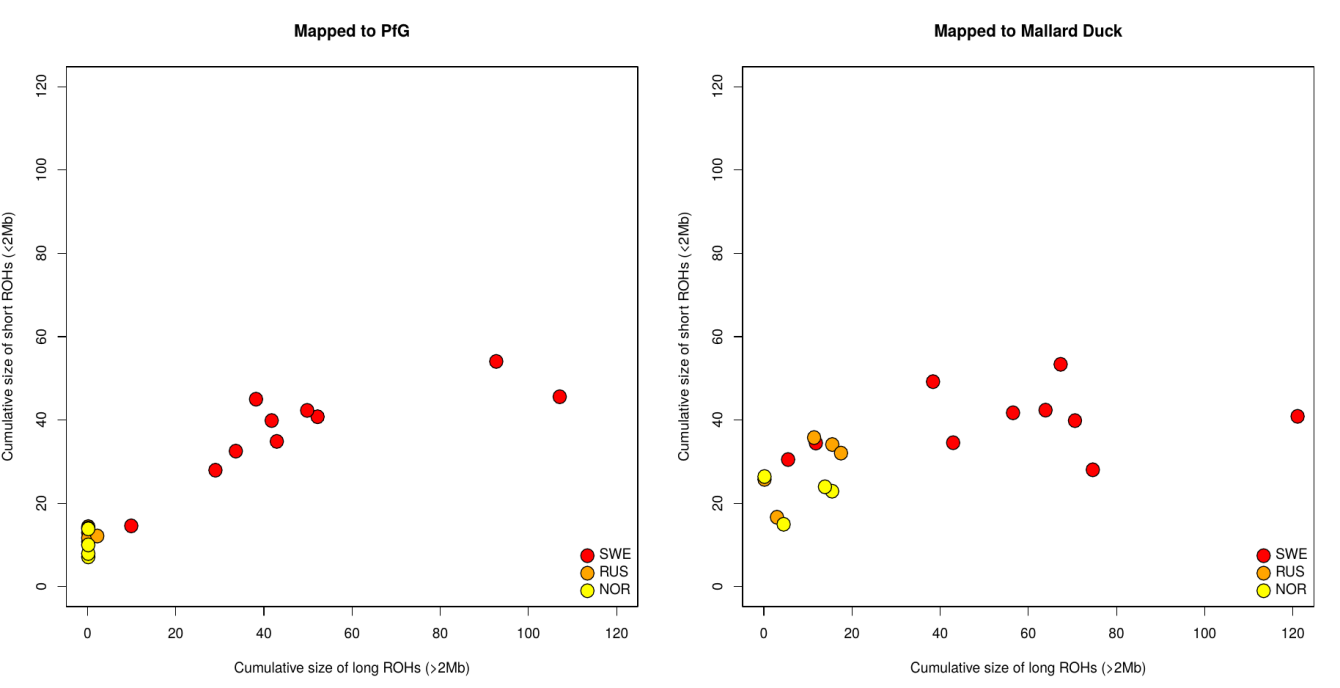


Figure S12. Cumulative inbreeding due to short (<2Mb) vs long (>2Mb) ROHs for all LWfG birds. Colors indicate different populations of origin.A) data mapped to the PfG. B) data mapped to the mallard duck.

**2.3 Introgression**

2.3.1 D-statistics

We used D-statistics, also known as ABBA-BABA tests [[1](https://paperpile.com/c/e1U4If/53vl9)6], to check whether there is evidence of GWfG introgression into the Swedish population of LWfG with respect to the Russian and Norwegian ones. The test requires of three samples H1, H2 and H3 that form a tree such as H1 and H2 being more related between them than to H3, and an outgroup O. This test is restricted to biallelic polymorphisms for which the outgroup carries one nucleotide (considered ‘ancestral allele’ and denoted as A), and H3 carries another nucleotide (considered ‘derived allele’ and denoted as B). We then call ABBA to variable positions for which H2 and H3 share the derived allele, and BABA to positions in which H1 and H3 share the derived allele. Thus, in a test D(H1, H2; H3, O) = (ABBA - BABA) / (ABBA+BABA), the same number of ABBA and BABA sites is expected (D=0) in the absence of genetic affinities or admixtures between H3 and either H1 or H2. A positive D (D>0) would indicate more affinity than expected between H2 and H3, and a negative D (D<0), affinity between H1 and H3.

To estimate GWfG introgression in SWE LWfG samples with respect to NOR and RUS we performed tests of the form D(SWE, RUS/NOR; GWfG, PfG), where SWE are all possible Swedish LWfG birds, RUS and NOR are all possible Russian and Norwegian birds respectively, the GWfG sample is used as donor, and the pink-footed goose as outgroup since this species was used as reference and also sits outside of the variation of GWfG and LWfG [[4](https://paperpile.com/c/e1U4If/MZtSP),5]. We used popstats [[1](https://paperpile.com/c/e1U4If/KkfqB)7] to perform all D-statistic tests on the filtered variant calls, sampling one allele at random per site [[1](https://paperpile.com/c/e1U4If/53vl9)6]. We repeated the analysis with the data mapped to the mallard duck. Significance of any deviation from 0 was assessed using a Z-score based on jackknife resampling of only 1Mb block size on the tests using the data mapped to PfG (due to the low contiguity of the assembly) and 5Mb when using the data mapped to the mallard duck.

Additionally, to explore evidence of introgression from all the other *Anser* species into the Swedish LWfG population respect to the Russian and Norwegian populations, we used tests of the form D(SWE, RUS/NOR; *Anser*, BhG), where SWE are all possible Swedish LWfG birds, RUS and NOR are all possible Russian and Norwegian birds respectively, all possible *Anser sp.* samples are used as donors, and the bar-headed goose as outgroup since this species sits outside of all the variation of *Anser* [5]. We used the same settings as above for all tests and replicated them using both the data mapped to the PfG assembly and to the mallard duck assembly.

Our results suggest that there is not an excess of introgression from GWfG into the Swedish birds with respect to the Norwegian or Russian ones (Figure 2). The same result is evident also in the dataset mapped to the mallard duck (Figure S13). We find very similar results when testing introgression from GWfG into LWfG using the bar-headed goose as outgroup for both datasets, the one mapped to PfG (Figure S14A) and the one mapped to the mallard duck (Figure S14B). Finally, none of the tests using other *Anser* species as donors deviated from zero for the data mapped to PfG or mallard duck (Figure S15), suggesting no introgression into Swedish LWfG birds relative to the Norwegian or Russian ones from these species.


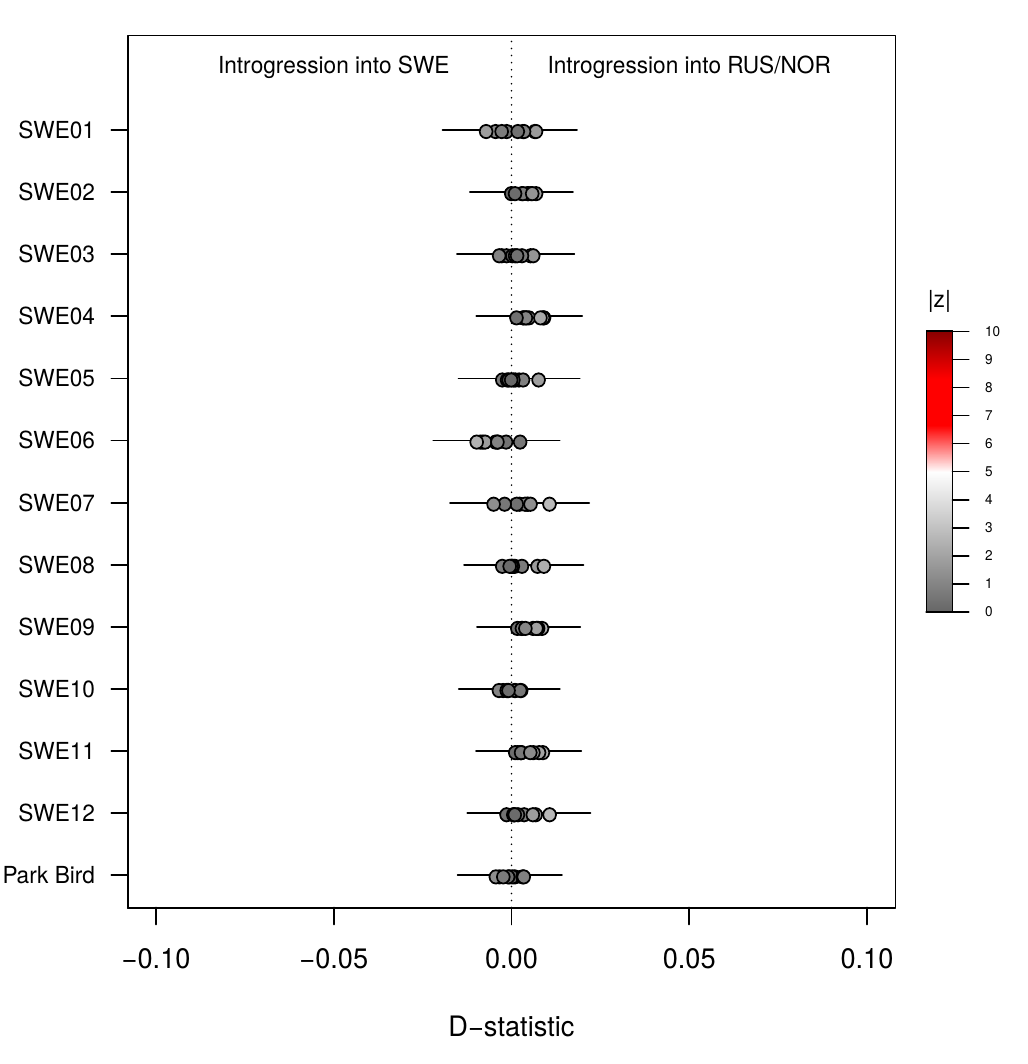


Figure S13. Estimates of introgression from GWfG into SWE and RUS/NOR LWfG individuals using PfG as outgroup and data mapped to the mallard duck. The tests are in the form D(SWE, RUS/NOR, GWfG, PfG). Error bars depicting 3*SE are displayed. Dots are colored according the |z| value of the test.


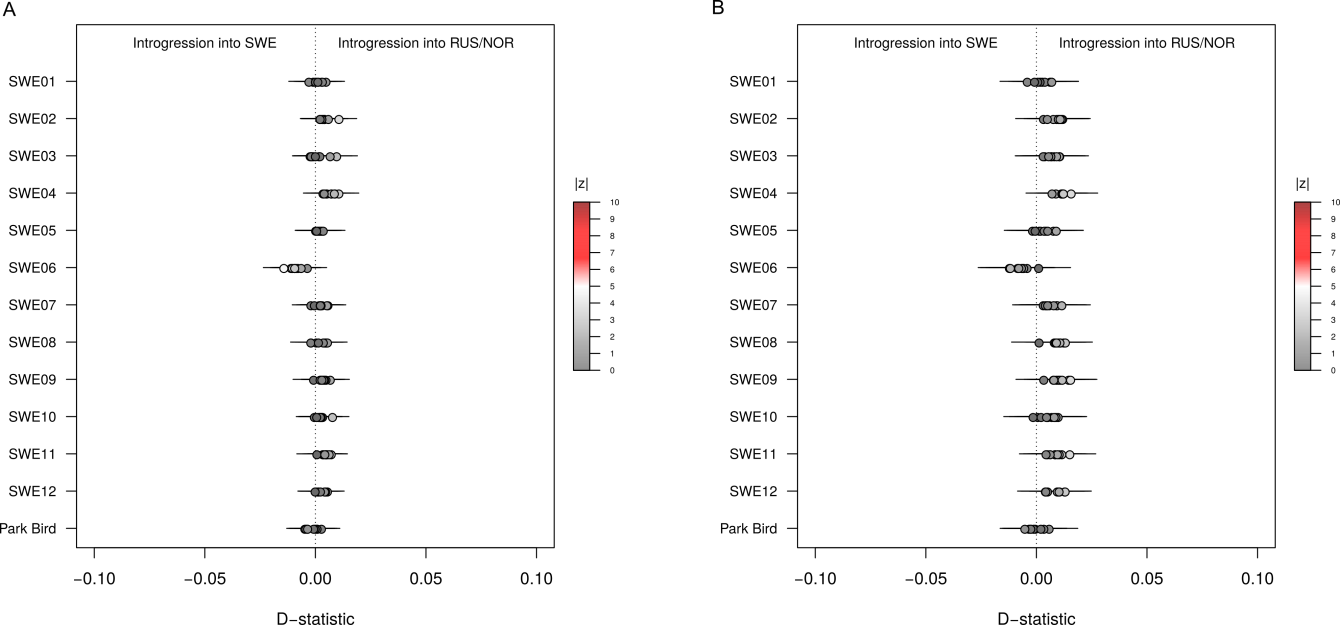


Figure S14. Estimates of introgression from GWfG into SWE and RUS/NOR LWfG individuals using bar-headed goose as outgroup. The tests are in the form D(SWE, RUS/NOR; GWfG, BhG). Error bars represent 3*SE. Dots are colored according the |z| value of the test. A) Data mapped to the PfG. B) Data mapped to the mallard duck.


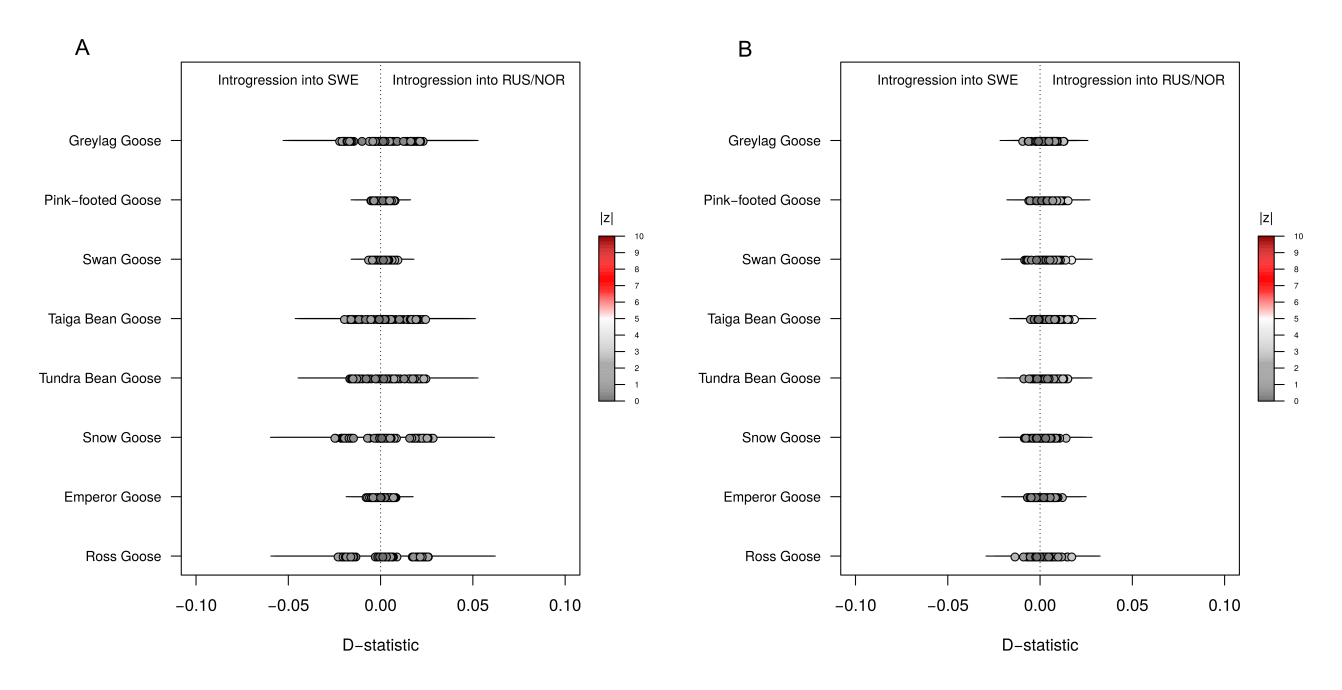


Figure S15. Estimates of introgression from eight *Anser sp*. into SWE and RUS/NOR LWfG individuals using bar-headed goose as outgroup. The tests are in the form D(SWE, RUS/NOR, *Anser sp*., BhG). Error bars represent 3*SE. Dots are colored according the |z| value of the test. A) Data mapped to the PfG. B) Data mapped to the mallard duck.

2.3.2 Probability of detecting introgression

In order to assess whether or not finding no introgression could be a sampling artifact, we calculated the probability of having missed individuals with GWfG ancestry in our 10 individuals sampled in 2010, the largest batch of LWfG samples in the dataset.

To do this, we first estimated the expected proportion ($f$) of LWfG birds with GWfG ancestry in the wild in 2010. Assuming an initial frequency of LWfG individuals with GWfG ancestry (*X*), the expected proportion of introgressed LWfG individuals *n* generations later, assuming no selective disadvantage for those with hybrid ancestry, can be estimated using the equation $f =1 - {(1-X)}^{2^{n}}$. We assumed that the initial proportion of birds with introgressed genes was 5%, which is a conservatively low number given that the breeding program was estimated to have released 5-10 % birds containing GWfG introgressed genes [18]. To estimate the number of generations passed until 2010, we assumed a generation time of 3 to 5 years, based on a recent study on pink-footed geese (2) and unpublished life-history data (5 years; N. Liljebäck, pers. obs). Using the mid-point of the reinforcement program (1990), this resulted in 4-6 LWfG generations having passed by the year 2010. Using these values we estimated the expected proportion of individuals with introgressed GWfG genes in 2010 as $f = 0.56-0.96$.

Second, we apply a hypergeometric distribution and perform a sampling of individuals without replacement analysis [19] to estimate the probability of not detecting any introgressed individuals in our sample of 10 birds from a finite population of size 110. Assuming $f = 0.56 and 0.96$, the probability of not detecting any introgressed birds in our sample is P < 0.001 and P < 0.0001, respectively (Figure S16). Consequently, it is very unlikely that any meaningful GWfG ancestry had gone undetected given our sampling effort.


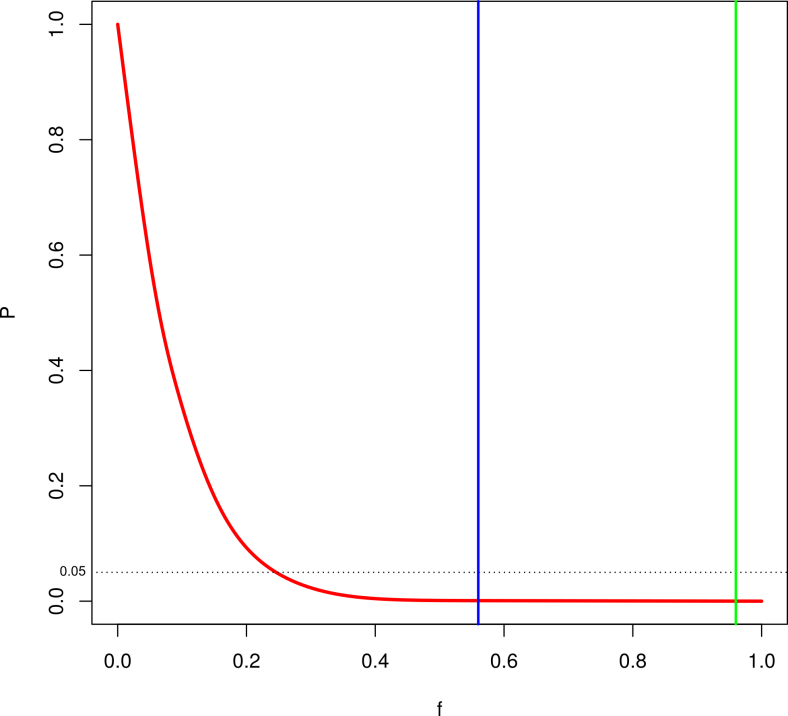


Figure S16. Distribution of probabilities of LWfG hybrid birds escaping detection given their frequency in the population based on sampling without replacement. The x-axis represents all possible frequencies of hybrid descendants in the population (*f*), and the y-axis, the associated probabilities of not detecting any hybrids (P) in a sample of 10 individuals from a finite population of 110 individuals. P = 0.05 is represented with a dotted line. The green and blue lines represent the estimated *f* of 0.56 and 0.96 for LWfG in 2010 (see text).

2.3.3. Mitochondrial analyses

In the late 90s, a series of studies based on mitochondrial DNA data, suggested that the LWfG captive population carried introgressed GWfG haplotypes [20]. We then used the shotgun sequencing data to reconstruct the complete mitogenomes of our LWfG samples and investigate possible introgression from GWfG.

There are currently no published complete mitogenomes for either LWfG or PfG, therefore, we used the mitogenome of a GWfG (NC_004539.1) as reference. Briefly, we mapped the raw reads to the reference using *BWA mem*, excluded PCR duplicates using *samtools rmdup* and generated consensus FASTA files with *ANGSD -dofasta* [21], using a majority rule, excluding reads with <30 mapping quality, sites with <30 base quality, and sites covered for less than 100 reads. We then performed multiple alignment of the reconstructed LWfG mitogenomes together with the previously published mitogenomes of two bar-headed goose and four GWfG in MAFFT [22]. Finally, we built a ML tree using the GTR model in MEGAX [23] with 100 bootstrap replicates.

We find that all LWfG mitogenomes formed a monophyletic clade that was separate from the GWfG mitogenomes, showing that no wild LWfG in our study carry GWfG mitogenomes, consistent with no admixture between these species (Figure S17A).


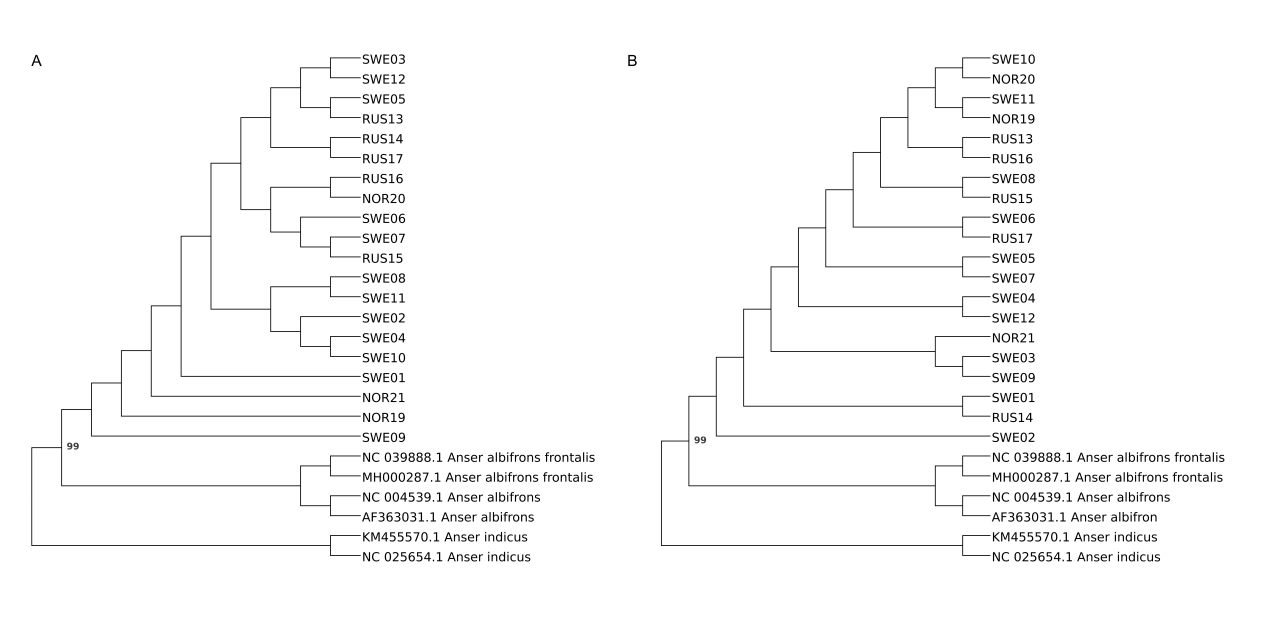


Figure S17. Maximum-likelihood trees of geese mitogenomes. Bootstrap values for the split between GWfG and LWfG samples is shown. The tree was rooted in the two bar-headed goose samples. A) all sites. B) excluding sites detected as variable.

However, when carefully inspecting the reconstructed mitogenomes we detected several sites that appeared variable. That is, sites with a similar number of reads with two different alleles. Since this is likely caused by nuclear insertions of mitochondrial DNA (i.e. numts), we sought to identify and exclude all these sites from the alignment. To identify these variable positions we called variants along the mitogenomes of all LWfG samples using *samtools mpileup*, excluding reads with <30 mapping quality, sites with <30 base quality, and sites covered for less than 100 reads. We identified 37 variable positions across individuals. After excluding these from the alignment, we constructed a ML tree as above. Again, all LWfG formed a monophyletic clade to the exclusion of GWfG, consistent with a lack of admixture from GWfG into LWfG (Figure S17B).

Since these variable positions are distributed all along the mitogenome, and we did not identify a specific region of the mitogenome with higher coverage than the rest, we hypothesize that the nuclear genomes of goose contain numts spanning the whole mitogenome sequence. Thus, identifying the numt sequences is critical to accurately reconstruct mitogenomes from shotgun sequencing data in future studies on goose.

**3. Supplementary references**

1. [Li, H. & Durbin, R. Fast and accurate short read alignment with Burrows–Wheeler transform.](http://paperpile.com/b/e1U4If/O8a18) *[Bioinformatics](http://paperpile.com/b/e1U4If/O8a18)***[25](http://paperpile.com/b/e1U4If/O8a18)**[, 1754–1760 (2009).](http://paperpile.com/b/e1U4If/O8a18)

2. [Pujolar, J. M., Dalén, L., Olsen, R. A., Hansen, M. M. & Madsen, J. First de novo whole genome sequencing and assembly of the pink-footed goose.](http://paperpile.com/b/e1U4If/wSk86) *[Genomics](http://paperpile.com/b/e1U4If/wSk86)***[110](http://paperpile.com/b/e1U4If/wSk86)**[, 75–79 (2018).](http://paperpile.com/b/e1U4If/wSk86)

3. [Huang, Y.](http://paperpile.com/b/e1U4If/MHgt4) *[et al.](http://paperpile.com/b/e1U4If/MHgt4)* [The duck genome and transcriptome provide insight into an avian influenza virus reservoir species.](http://paperpile.com/b/e1U4If/MHgt4) *[Nat. Genet.](http://paperpile.com/b/e1U4If/MHgt4)***[45](http://paperpile.com/b/e1U4If/MHgt4)**[, 776–783 (2013).](http://paperpile.com/b/e1U4If/MHgt4)

4. [Ottenburghs, J.](http://paperpile.com/b/e1U4If/MZtSP) *[et al.](http://paperpile.com/b/e1U4If/MZtSP)* [A tree of geese: A phylogenomic perspective on the evolutionary history of True Geese.](http://paperpile.com/b/e1U4If/MZtSP) *[Mol. Phylogenet. Evol.](http://paperpile.com/b/e1U4If/MZtSP)***[101](http://paperpile.com/b/e1U4If/MZtSP)**[, 303–313 (2016).](http://paperpile.com/b/e1U4If/MZtSP)

5. [Ottenburghs, J.](http://paperpile.com/b/ei2Iad/Oywga) *[et al.](http://paperpile.com/b/ei2Iad/Oywga)* [A history of hybrids? Genomic patterns of introgression in the True Geese.](http://paperpile.com/b/ei2Iad/Oywga) *[BMC Evol. Biol.](http://paperpile.com/b/ei2Iad/Oywga)***[17](http://paperpile.com/b/ei2Iad/Oywga)**[, 201 (2017).](http://paperpile.com/b/ei2Iad/Oywga)

6. [Smit, A. F. A., Hubley, R. & Green, P. 1996--2010. RepeatMasker Open-3.0. (2017).](http://paperpile.com/b/e1U4If/imyEC)

7. [Smit, A. & Hubley, R. RepeatModeler Open-1.0 (2015).](http://paperpile.com/b/e1U4If/woqQE)

8. [Li, H. A statistical framework for SNP calling, mutation discovery, association mapping and population genetical parameter estimation from sequencing data.](http://paperpile.com/b/e1U4If/ysFeo) *[Bioinformatics](http://paperpile.com/b/e1U4If/ysFeo)***[27](http://paperpile.com/b/e1U4If/ysFeo)**[, 2987–2993 (2011).](http://paperpile.com/b/e1U4If/ysFeo)

9. [Pečnerová, P.](http://paperpile.com/b/e1U4If/EchjR) *[et al.](http://paperpile.com/b/e1U4If/EchjR)* [Genome-Based Sexing Provides Clues about Behavior and Social Structure in the Woolly Mammoth.](http://paperpile.com/b/e1U4If/EchjR) *[Curr. Biol.](http://paperpile.com/b/e1U4If/EchjR)***[27](http://paperpile.com/b/e1U4If/EchjR)**[, 3505–3510.e3 (2017).](http://paperpile.com/b/e1U4If/EchjR)

10. [Danecek, P.](http://paperpile.com/b/e1U4If/P6uIp) *[et al.](http://paperpile.com/b/e1U4If/P6uIp)* [The variant call format and VCFtools.](http://paperpile.com/b/e1U4If/P6uIp) *[Bioinformatics](http://paperpile.com/b/e1U4If/P6uIp)***[27](http://paperpile.com/b/e1U4If/P6uIp)**[, 2156–2158 (2011).](http://paperpile.com/b/e1U4If/P6uIp)

11. [Manichaikul, A.](http://paperpile.com/b/e1U4If/88z9A) *[et al.](http://paperpile.com/b/e1U4If/88z9A)* [Robust relationship inference in genome-wide association studies.](http://paperpile.com/b/e1U4If/88z9A) *[Bioinformatics](http://paperpile.com/b/e1U4If/88z9A)***[26](http://paperpile.com/b/e1U4If/88z9A)**[, 2867–2873 (2010).](http://paperpile.com/b/e1U4If/88z9A)

12. [Weir, B. S. & Cockerham, C. C. Estimating f-statistics for the analysis of population structure.](http://paperpile.com/b/e1U4If/CMtNA) *[Evolution](http://paperpile.com/b/e1U4If/CMtNA)***[38](http://paperpile.com/b/e1U4If/CMtNA)**[, 1358–1370 (1984).](http://paperpile.com/b/e1U4If/CMtNA)

13. [Pickrell, J. K. & Pritchard, J. K. Inference of Population Splits and Mixtures from Genome-Wide Allele Frequency Data.](http://paperpile.com/b/e1U4If/GXhS6) *[PLoS Genet.](http://paperpile.com/b/e1U4If/GXhS6)***[8](http://paperpile.com/b/e1U4If/GXhS6)**[, e1002967 (2012).](http://paperpile.com/b/e1U4If/GXhS6)

14. [Pemberton, T. J.](http://paperpile.com/b/e1U4If/VEVhO) *[et al.](http://paperpile.com/b/e1U4If/VEVhO)* [Genomic Patterns of Homozygosity in Worldwide Human Populations.](http://paperpile.com/b/e1U4If/VEVhO) *[Am. J. Hum. Genet.](http://paperpile.com/b/e1U4If/VEVhO)***[91](http://paperpile.com/b/e1U4If/VEVhO)**[, 275–292 (2012).](http://paperpile.com/b/e1U4If/VEVhO)

15 [Purcell, S.](http://paperpile.com/b/e1U4If/Y3YKa) *[et al.](http://paperpile.com/b/e1U4If/Y3YKa)* [PLINK: a tool set for whole-genome association and population-based linkage analyses.](http://paperpile.com/b/e1U4If/Y3YKa) *[Am. J. Hum. Genet.](http://paperpile.com/b/e1U4If/Y3YKa)***[81](http://paperpile.com/b/e1U4If/Y3YKa)**[, 559–575 (2007).](http://paperpile.com/b/e1U4If/Y3YKa)

16. [Green, R. E.](http://paperpile.com/b/e1U4If/53vl9) *[et al.](http://paperpile.com/b/e1U4If/53vl9)* [A Draft Sequence of the Neandertal Genome.](http://paperpile.com/b/e1U4If/53vl9) *[Science](http://paperpile.com/b/e1U4If/53vl9)***[328](http://paperpile.com/b/e1U4If/53vl9)**[, 710–722 (2010).](http://paperpile.com/b/e1U4If/53vl9)

17. Skoglund, P. *et al.* Genetic evidence for two founding populations of the Americas, *Nature* **525**, 104-108 (2015).

18. [Naturvårdsverket.](http://paperpile.com/b/e1U4If/pMHcV) *[Åtgärdsprogram för fjällgås 2011–2015](http://paperpile.com/b/e1U4If/pMHcV)*[. (Naturvårdsverket, 2011).](http://paperpile.com/b/e1U4If/pMHcV)

19. [Sjogren, P. & Wyoni, P.-I. Conservation Genetics and Detection of Rare Alleles in Finite Populations.](http://paperpile.com/b/e1U4If/MLxQb) *[Conserv. Biol.](http://paperpile.com/b/e1U4If/MLxQb)***[8](http://paperpile.com/b/e1U4If/MLxQb)**[, 267–270 (1994).](http://paperpile.com/b/e1U4If/MLxQb)

20. [Ruokonen, M., Kvist, L., Tegelström, H. & Lumme, J. Goose hybrids, captive breeding and restocking of the Fennoscandian populations of the Lesser White-fronted goose (](http://paperpile.com/b/ei2Iad/TYEZk)*[Anser erythropus](http://paperpile.com/b/ei2Iad/TYEZk)*[).](http://paperpile.com/b/ei2Iad/TYEZk) *[Conserv. Genet.](http://paperpile.com/b/ei2Iad/TYEZk)***[1](http://paperpile.com/b/ei2Iad/TYEZk)**[, 277–283 (2000).](http://paperpile.com/b/ei2Iad/TYEZk)

21. [Korneliussen, T. S., Albrechtsen, A. & Nielsen, R. ANGSD: Analysis of Next Generation Sequencing Data.](http://paperpile.com/b/e1U4If/KkfqB) *[BMC Bioinformatics](http://paperpile.com/b/e1U4If/KkfqB)***[15](http://paperpile.com/b/e1U4If/KkfqB)**[, 356 (2014).](http://paperpile.com/b/e1U4If/KkfqB)

22. Katoh, K., Standley, D.M. MAFFT multiple sequence alignment software version 7: improvements in performance and usability. *Mol. Biol. Evol.* **30**, 772-80 (2013)

23. Kumar, S., Stecher, G., Li, M., Knyaz, C., Tamura, K. MEGA X: molecular evolutionary genetics analysis across computing platforms. *Mol. Biol. Evol.* **35**, 1547-9 (2018).
